# Secondary Metabolites Profiled in Cannabis Inflorescences, Leaves, Stem Barks, and Roots for Medicinal Purposes

**DOI:** 10.1038/s41598-020-60172-6

**Published:** 2020-02-24

**Authors:** Dan Jin, Kaiping Dai, Zhen Xie, Jie Chen

**Affiliations:** 1 Biomedical Engineering Department, University of Alberta, Edmonton, Alberta Canada; 2 Labs-Mart Inc., Edmonton, Alberta Canada; 3 Electrical and Computer Engineering Department, University of Alberta, Edmonton, Alberta Canada

**Keywords:** Secondary metabolism, Analytical chemistry

## Abstract

Cannabis research has historically focused on the most prevalent cannabinoids. However, extracts with a broad spectrum of secondary metabolites may have increased efficacy and decreased adverse effects compared to cannabinoids in isolation. Cannabis’s complexity contributes to the length and breadth of its historical usage, including the individual application of the leaves, stem barks, and roots, for which modern research has not fully developed its therapeutic potential. This study is the first attempt to profile secondary metabolites groups in individual plant parts comprehensively. We profiled 14 cannabinoids, 47 terpenoids (29 monoterpenoids, 15 sesquiterpenoids, and 3 triterpenoids), 3 sterols, and 7 flavonoids in cannabis flowers, leaves, stem barks, and roots in three chemovars available. Cannabis inflorescence was characterized by cannabinoids (15.77–20.37%), terpenoids (1.28–2.14%), and flavonoids (0.07–0.14%); the leaf by cannabinoids (1.10–2.10%), terpenoids (0.13–0.28%), and flavonoids (0.34–0.44%); stem barks by sterols (0.07–0.08%) and triterpenoids (0.05–0.15%); roots by sterols (0.06–0.09%) and triterpenoids (0.13–0.24%). This comprehensive profile of bioactive compounds can form a baseline of reference values useful for research and clinical studies to understand the “entourage effect” of cannabis as a whole, and also to rediscover therapeutic potential for each part of cannabis from their traditional use by applying modern scientific methodologies.

## Introduction

**Figure 1:**
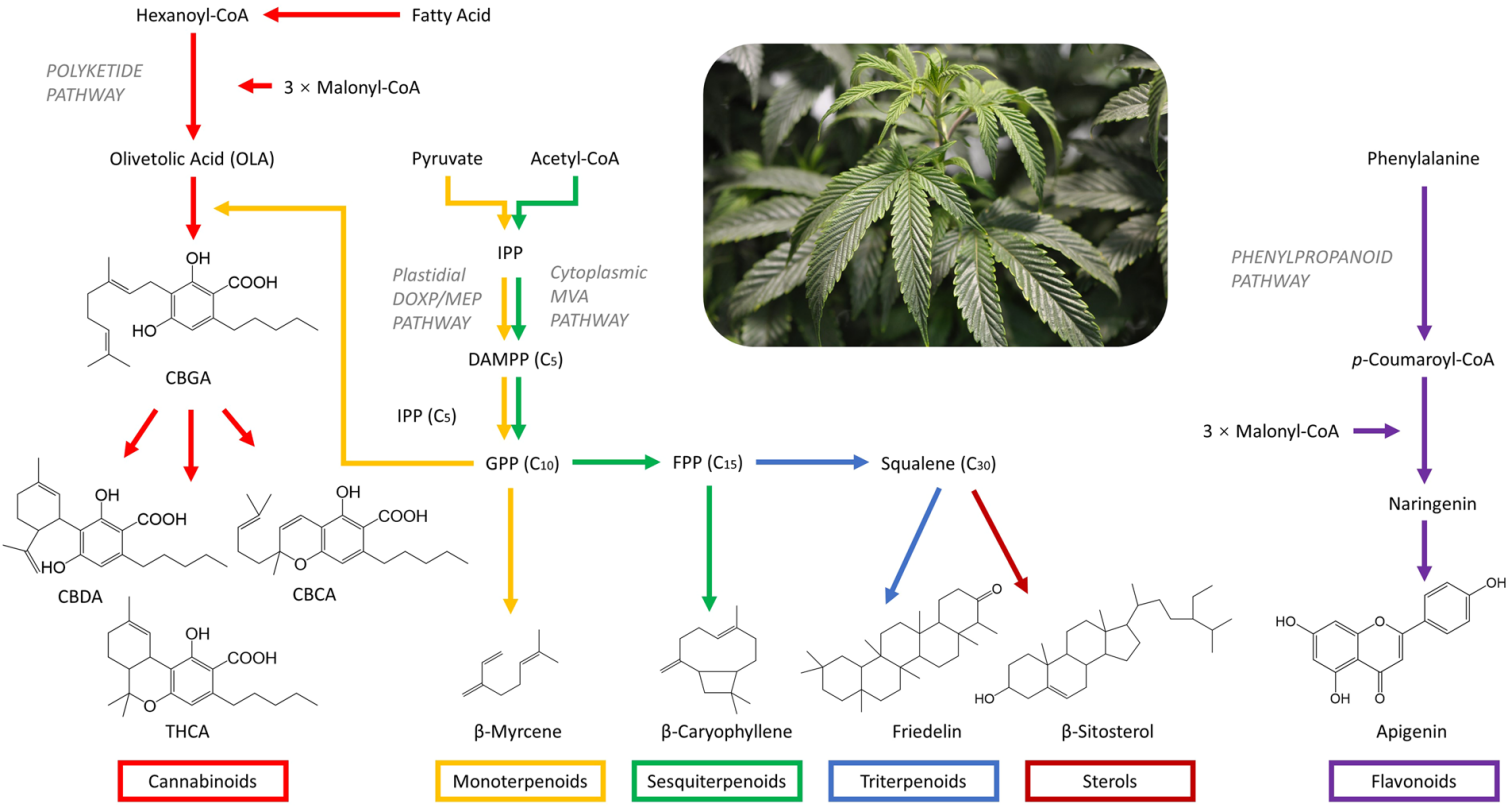
Biosynthesis pathways of cannabinoid, terpenoids, sterols, and flavonoids^7,8^. Cannabinoids and terpenoids are produced and stored in the secretory cells of glandular trichomes, which are found in the aerial parts of cannabis plants and are especially dense on the top surfaces of seedless female flowers^38^. Two precursors for cannabinoids are olivetolic acid (OLA), derived from the polyketide pathway, and geranyl diphosphate (GPP), derived from the plastidial deoxyxylulose phosphate/methyl-erythritol phosphate (DOXP/MEP pathway)^102–104^. Cannabigerolic acid (CBGA) is formed by the condensation of OLA and GPP and is further converted to cannabidiolic acid (CBDA), ∆^9^-tetrahydrocannabinolic acid (∆^9^-THCA), and cannabichromenic acid (CBCA) by CBDA synthase^105^, ∆^9^-THCA synthase^106^, and CBCA synthase^107^, respectively. If divarinic acid is condensed with GPP instead of OLA, the propyl (C_3_ side-chain) instead of pentyl (C_5_ side-chain) cannabigerovarinic acid (CBGVA) is produced, which can be further converted to cannabidivarinic acid (CBDVA), tetrahydrocannabivarinic acid (THCVA), and cannabichromevarinic acid (CBCVA) following similar pathways^53^. Terpenoids are derived from the mevalonate (MVA) pathway or from the DOXP/MEP pathway. Both pathways produce isopentenyl diphosphate (IPP), which is further isomerized to dimethylallyl diphosphate (DMAPP), at their endpoints^53^. The DOXP/MEP pathway provides GPP to form monoterpenoids (C_10_) while MVA pathway provides farnesyl diphosphate (FPP) for sesquiterpenoids (C_15_) and squalene as precursors for triterpenoids (C_30_) and sterols^53^. Flavonoids in cannabis, mainly flavones (luteolin, apigenin, orientin, vitexin, and isovitexin) and flavonols (quercetin and kaempferol), exist as free aglycones or as conjugated *O*-glycosides or *C*-glycosides^7,52,108,109^. The phenylpropanoid pathway produces *p*-coumaroyl-CoA from phenylalanine. In conjunction with three molecules of malonyl-CoA, *p*-coumaroyl-CoA produces naringenin, which is the substrate for flavone and flavonol biosynthesis^8,53^.

Cannabis is a complex herbal medicine containing several classes of secondary metabolites, including at least 104 cannabinoids, 120 terpenoids (including 61 monoterpenes, 52 sesquiterpenoids, and 5 triterpenoids), 26 flavonoids, and 11 steroids among 545 identified compounds^1–6^. The postulated biosynthetic pathways for these metabolite groups^7,8^ are outlined in Fig. 1. Cannabis has attracted a new wave of interest for its broad medicinal applications as 1) an analgesic, potentially as an adjunct to or substitute for opiates in the treatment of chronic pain^9^, and 2) an appetite stimulant and digestive aid^10^, among others. Since the 1960s, the research has focussed mainly on cannabinoids, ∆^9^-tetrahydrocannabinol (∆^9^-THC), and cannabidiol (CBD) in particular^11–28^. The major psychoactive content expressed as total THC decreases in the order of inflorescences (10–12%), leaves (1–2%), stems (0.1–0.3%), roots (<0.03%), and seeds (generally absent)^29^. As such, female flower tops are harvested while other parts are often discarded by growers^29^. This is a potentially unnecessary waste. As an ancient medicine in various cultures, each part of the cannabis plant has been historically indicated with a wide range of applications relating mostly to painkilling, inflammation releasing, and mental illness treatment^30–33^.

Compounds other than ∆^9^-THC and CBD may contribute to the therapeutic effects of each plant part in their traditional uses. Minor cannabinoids, such as cannabinol (CBN), cannabigerol (CBG), cannabichromene (CBC), also have broad therapeutic potential^11,34–37^. Terpenoids may directly elicit physiological effects or modulate cannabinoid responses^38^. Flavonoids share a wide range of biological effects with cannabinoids and terpenoids that include anti-inflammatory, anti-cancer, and neuroprotective properties^39^. One of the triterpenoids identified in cannabis root, friedelin, contains anti-inflammatory, antioxidant, estrogenic, anti-cancer, and liver protectant properties^31^. Plant sterols may reduce plasma cholesterol levels^40–44^. The combination of different secondary metabolites of varying concentrations is believed to increase the range of therapeutic properties –known as the “entourage effect”^38,45,46^. One recent study showed that whole plant extracts are more beneficial than pure CBD for the treatment of inflammatory conditions in mice^47^. Another preclinical study has shown that a botanical cannabis preparation was more effective than pure THC in producing antitumor responses *in vitro*^45^. However, the increased potency was attributable to compounds other than the five most abundant terpenes in the preparation^45^. The literature suggests that a wider range of bioactive compounds should be included when examining the beneficial medicinal properties of botanical cannabis preparations.

**Figure 2:**
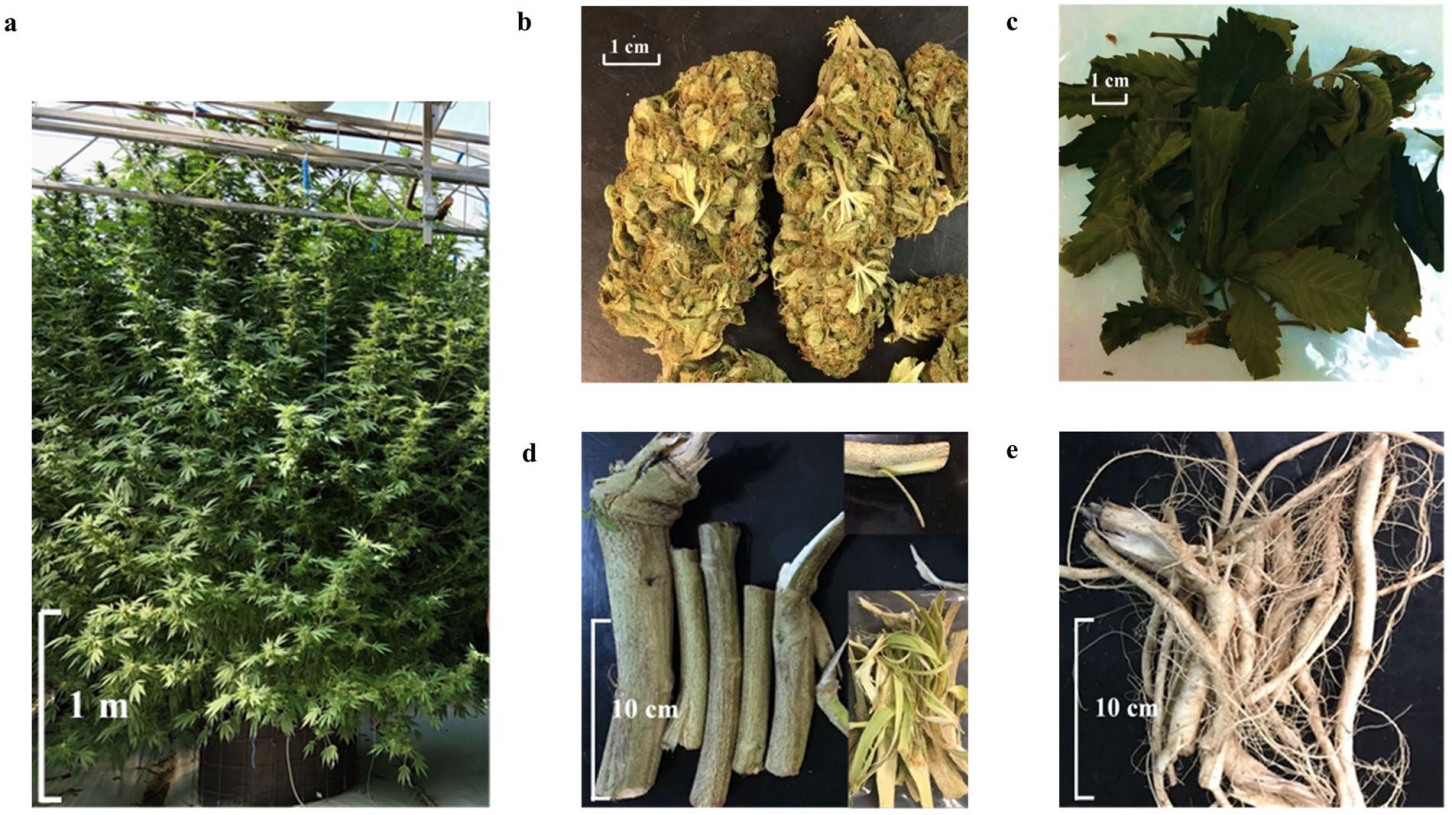
Cannabis CBD Mango Haze plant, inflorescences, leaves, root, stem barks, and roots. (**a**) CBD Mango Haze plant that has been kept in vegetative stage for six months and initiated flowering for two months in a greenhouse. (**b**) Dried cannabis inflorescences. (**c**) Dried cannabis leaves. (**d**) Fresh cannabis stems with barks and later peeled (right corner). (**e**) Fresh root material.

The aim of this study is to leverage a comprehensive investigation of chemical profiles in each plant part. The metabolites of the study included 14 cannabinoids, 47 terpenoids (29 monoterpenoids, 15 sesquiterpenoids, and 3 triterpenoids), 3 sterols, and 7 flavonoids. This multipart study included the development of quantitative methods using liquid chromatography coupled with mass spectroscopy (LC-MS) for cannabinoids, liquid chromatography coupled with a standard ultraviolet detector and mass spectroscopy (LC-UV-MS) for flavonoids, and gas chromatography coupled with mass spectroscopy (GC-MS) for terpenoids and sterols. Relevant compounds were selected based on their pharmacological activities^5,8^, or use in other cannabis classification studies^48–51^. The methods were then employed for generating the chemical profiles of the inflorescences, leaves, stem barks, and roots of three selected cannabis chemovars (Fig. 2). The results can form a baseline of reference values useful for future research and clinical studies on these compounds’ pharmacological activity.

## Results

### Sample preparation optimization

The yield of total cannabinoids averaged 17.5 ± 0.5% (n = 5) using manual grinding with a handheld herb grinder, which was significantly higher (n = 5, p < 0.0001) than using an electric blender, a yield of which averaged 12.0 ± 0.3%. The minimization of analyte loss using the manual grinder is attributed to the fact that resin adheres to the blades and plastic housing surface of a plastic blender during high-speed pulverization (Supplementary Fig. 1). There were no significant differences in extraction efficiency for cannabinoids between two solvents, methanol and a 9:1 methanol/chloroform mixture (n = 5, p = 0.6379). Because methanol is less toxic than methanol/chloroform, methanol was used as the solvent in the following tests. The duration of sonication (10, 20, and 30 minutes) had no significant differences in cannabinoid extraction (n = 5, p = 0.3351). However, yield after sonication was found to be slightly lower than maceration for one day (n = 5, p = 0.0248). Four extraction methods were tested for terpenoids (sonication at 10, 20, and 30 minutes and maceration for one day after sonication for 20 minutes) and found to have no significant differences in total mono- and sesquiterpenoids yield (n = 5, p = 0.9904). Sonication at room temperature (20 °C) extracted higher total cannabinoids compared to 30 °C and 50 °C (n = 5, p = 0.018). Whether extraction was performed once, twice, or thrice did not have significant effects on total cannabinoid yield (n = 5, p = 0.3995). For all the following experiments, cannabinoids and terpenoids were extracted once using methanol by sonication at room temperature for 20 minutes. For extraction of total sterols in stem barks, sonication for one hour, maceration for one, two, three, four, and five days were significantly different (n = 5, p < 0.0001) and the main differences were between sonication and maceration. The differences between sonication and maceration for the extraction of total sterols in root material were not significant (n = 5, p = 0.0661). For extraction of total triterpenoids in stem bark material, sonication for one hour, maceration for one, two, three, four, and five days were not significantly different (n = 5, p = 0.8001). The comparison between sonication and maceration for the extraction of total triterpenoids in root material achieved similar results (n = 5, p = 0.1221). Despite a previous study’s concern that large amounts of cannabinoids may interfere with flavonoid quantification^52^, the three situations compared in this study (no hexane wash, one hexane wash, and three hexane washes before acid hydrolysis) had no significant difference in leaf material (n = 3, p = 0.8701) and a reduction in flavonoid yield in inflorescence material (n = 3, p < 0.0001).

### Method validation results for cannabinoids

**Figure 3:**
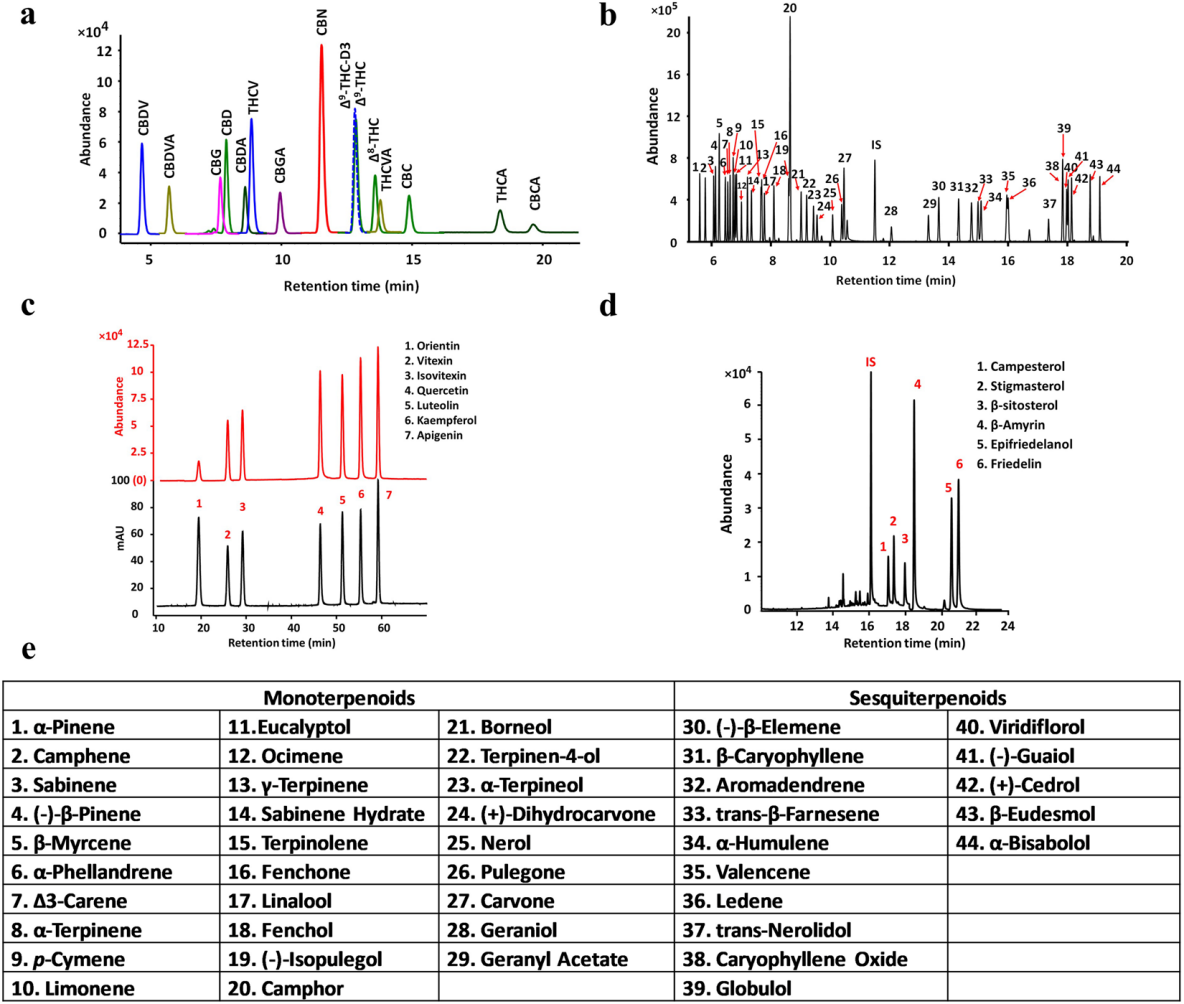
Chromatograms for cannabinoids, mono- and sesquiterpenoids, flavonoids, sterols, and triterpenoids. (**a**) Chromatogram for a standard solution of 14 mixed cannabinoids by LC-ESI-MS. (**b**) Chromatogram for 44 mono- and sesquiterpenoids by GC-MS. Terpenoids corresponded to the labeled number are listed in **e**. (**c**) Chromatogram for 7 flavonoids by HPLC-UV-MS. Mass spectrometry was used for flavonoid identification and UV detector was used for flavonoid quantification. (**d**) Chromatogram for 3 sterols and 3 triterpenoids by GC-MS. (**e**) Compound names for 44 mono- and sesquiterpenoids.

The chromatogram for a standard solution of 14 mixed cannabinoids by LC-MS is shown in Fig. 3a. The regression curves were found to be visibly linear, and the slope and coefficient of determination were calculated (Supplementary Table 1). The correlation coefficients for all 14 cannabinoids were above 0.9998. The intercept for each compound was set to zero because the p-value> 0.05 by analysis of variance (ANOVA) indicates insufficient evidence to reject the null hypothesis that the intercept is 0. The limit of detection (LOD) was between 0.0004 and 0.004 µg/mL, and the limit of quantification (LOQ) was between 0.001 and 0.01 µg/mL. Repeatability was between 0.4% and 9.2% for all compounds (Supplementary Table 2). Intermediate precision was between 1.5% and 12.3%. All relative biases were between −6.4% and 6.9% and all measurement uncertainties were between 1.5% and 12.3%. The matrix effect and extraction efficiency are listed in Supplementary Table 3. The matrix effect for all three levels was between 93.03–101.65%. The extraction recoveries for all three levels were between 80–120%, except for CBGA at 1.0 μg/mL (77.21%) and THCVA at 1.0 μg/mL (79.03%). Compared to their neutral forms, cannabinoid acids had higher degradation during sonication. Method robustness was verified using an alternate chromatographic column and a second LC-MS instrument. Neither the columns (n = 5, p = 0.2914) nor the machines (n = 5, p = 0.9580) showed significant differences in extracted cannabinoids.

### Method validation for mono- and sesquiterpenoids

The correlation coefficients for all 44 terpenoids were above 0.9989 (Supplementary Table 4). LOD were between 0.009 and 0.167 µg/mL, and LOQ were between 0.026 and 0.500 µg/mL. Repeatability was between 0.4% and 6.4% for all compounds (Supplementary Table 5). Intermediate precision was between 0.6% and 8.8%. All relative biases were between −6.3% and 8.7%, and all measurement uncertainties were between 1.5% and 9.1%. Robustness was evaluated by two analysts operating on the same machine by testing twelve replicate cannabis samples. Results were not significantly different in terms of total mono- and sesquiterpenoid yield (n = 5, p = 0.9588).

### Method validation for flavonoids

The correlation coefficients for all seven compounds were greater than 0.9997 (Supplementary Table 6). Trueness, determined by recovery, for seven flavonoids by acid hydrolysis were between 71.5 ± 1.3% and 106.6 ± 4.0% for level 1, between 70.5 ± 0.9% and 95.8 ± 0.8% for level 2, and between 75.1 ± 0.7% and 94.7 ± 1.7% for level 3 (Supplementary Table 7). Recovery for luteolin (84.1 ± 3.5% for level 1, 80.0 ± 2.6% for level 2, and 80.8 ± 1.5% for level 3) and apigenin (80.5 ± 0.9% for level 1, 78.7 ± 1.9% for level 2, and 81.1 ± 0.6%% for level 3) were comparable with a previous study’s recovery results of 82% for luteolin and 81% for apigenin^7^. The method is repeatable with intraday RSD% (n = 3) ranging between 1.20% and 4.10% for level 1, between 0.9% and 3.2% for level 2, and between 1.0% and 3.0% for level 3. The intermediate precision calculated from twelve replicates of leaf samples ranged between 1.70% and 3.3% and ranged between 2.1% and 5.6% for the cannabis inflorescence sample.

### Method validation for sterols and triterpenoids

The correlation coefficients for all 6 compounds were between 0.9989 and 0.9999 (Supplementary Table 8). LOD were between 0.17 and 0.26 µg/mL, and LOQ were between 0.50 and 0.79 µg/mL. Repeatability was between 0.4% and 9.2% for all compounds (Supplementary Table 9). Intermediate precision for 9 replicates was between 1.1% and 4.7%. All relative biases were between −4.0% and 1.4%, and all measurement uncertainties were between 1.4% and 5.8%.

### Cannabinoids profile in inflorescences, leaves, stem barks, and roots

**Figure 4:**
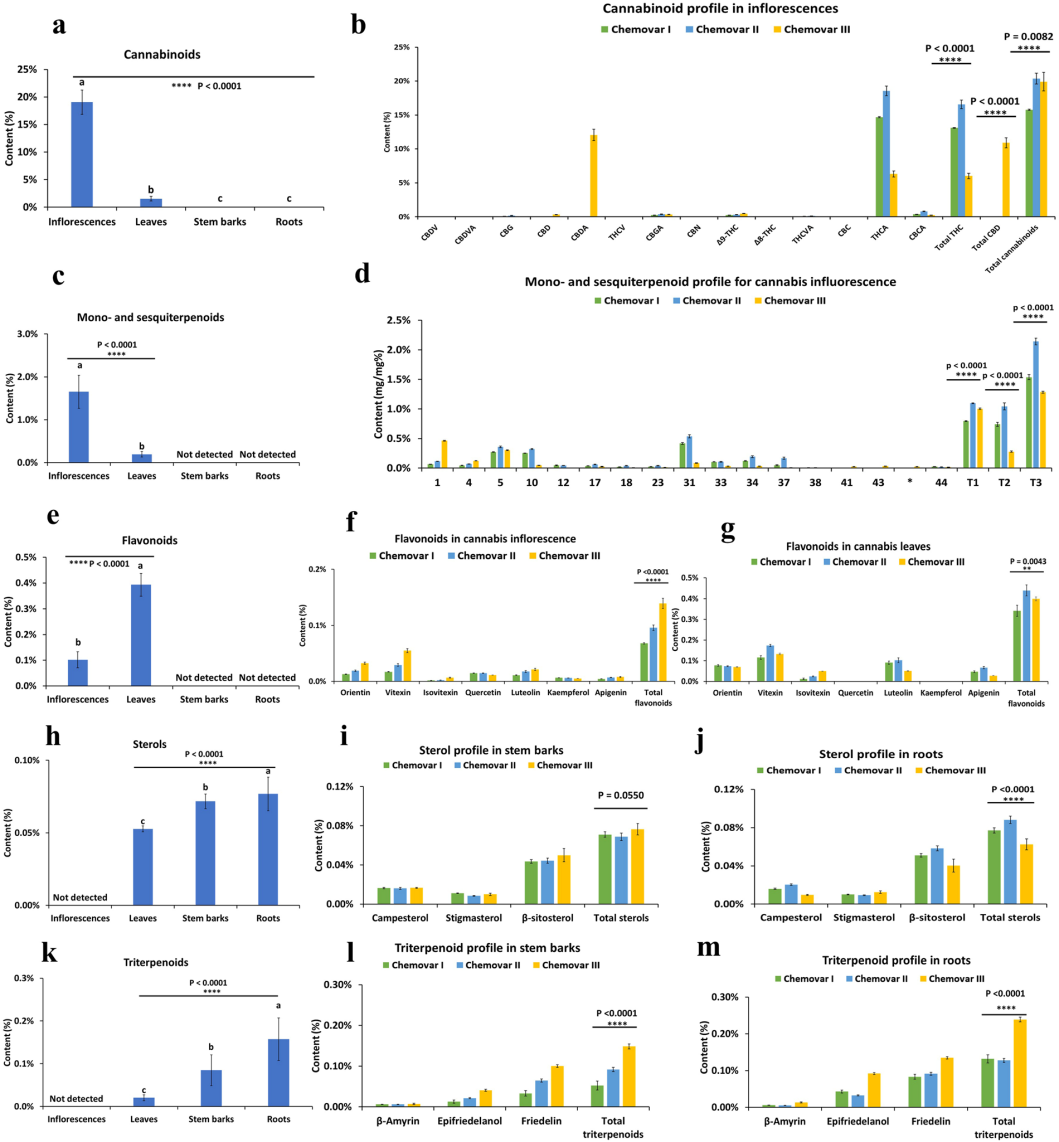
Secondary metabolites profiling in cannabis roots, stem barks, leaves, and inflorescences. (**a**) Total cannabinoid content (mg/mg%) in each part of cannabis plant averaged from three cannabis chemovars (n = 9, mean ± SD %). (**b**) Individual and total cannabinoid content (mg/mg%) in cannabis inflorescences of three chemovars (n = 3, mean ± SD %). Asterisks indicate statistically significant differences (one-way ANOVA, *p  <  0.05, **p  <  0.01, ***p  <  0.001, ****p  <  0.0001). (**c**) Total mono- and sesquiterpenoid content (mg/mg%) in each part of cannabis plant averaged from three cannabis chemovars (n = 9, mean ± SD %). (**d**) Individual and total mono- and sesquiterpenoids content (mg/mg%) in cannabis inflorescences of three chemovars (n = 3, mean ± SD %). Terpenoids labelled by their numbers are listed in Fig. 3e. The compound labelled as Asterisks was α-eudesmol, which was semi-quantified by GC-FID. T1 = total monoterpenoids. T2 = total sesquiterpenoids. T3 = total mono- and sesquiterpenoids. (**e**) Total flavonoid content (mg/mg%) in each part of cannabis plants averaged from three cannabis chemovars (n = 9, mean ± SD %). (**f**) Individual and total flavonoid content (mg/mg%) in cannabis inflorescences of three chemovars (n = 3, mean ± SD %). (**g**) Individual and total flavonoid content (mg/mg%) in cannabis leaves of three chemovars (n = 3, mean ± SD %). (**h**) Total sterol content (mg/mg%) in each part of cannabis plant averaged from three cannabis chemovars (n = 9, mean ± SD %). (**i**) Individual sterol content (mg/mg%) in cannabis stem barks of three chemovars (n = 3, mean ± SD %). (**j**) Individual sterol content (mg/mg%) in cannabis roots of three chemovars (n=3, mean ± SD %). (**k**) Total triterpenoid content (mg/mg%) in each part of cannabis plant averaged from three cannabis chemovars (n = 9, mean ± SD %). (**l**) Individual and total triterpenoid content (mg/mg%) in cannabis stem barks of three chemovars (n = 3, mean ± SD %). (**m**) Individual and total triterpenoid content (mg/mg%) in cannabis stem barks of three chemovars (n = 3, mean ± SD %). In each figure, the one-way ANOVA followed by correction for multiple comparisons (Tukey honestly significant difference (HSD) post hoc test) at the 0.05 significance level was used (p values indicated above each bar). Asterisks indicate statistically significant differences (one-way ANOVA, *p  <  0.05, **p  <  0.01, ***p  <  0.001, ****p  <  0.0001).

Cannabinoid content decreased from inflorescences to leaves, stem barks, and roots (Fig. 4a). Roots contained between 0.001% and 0.004% cannabinoids in all three chemovars (Supplementary Table 10), which agrees with the minuscule amounts reported by other studies (0% and 0.03%)^5,29^. Stem barks contained between 0.005% and 0.008% cannabinoids in all three chemovars and was found to be less than the amounts previously reported (0.02% and 0.1–0.3%)^5,29^. Differences may be caused by variations in chemovar and the position where the sample was taken (next to root). Cannabinoids quantified in cannabis leaf and inflorescence are shown in Fig. 4b and listed in Supplementary Table 11. Total cannabinoids quantified in leaves were between 1.10% and 2.10%, which agreed with the previously-reported amounts (1–2% and 1.40–1.75%)^29,53^ but not others (0.05%)^5^. Total cannabinoids quantified in inflorescence were between 15.77% and 20.37% in all three chemovars, as typical of modern drug-type chemovars^4,49,54,55^.

Chemovars I and II displayed THC dominant profiles, with THCA as the dominant compound (14.68% and 18.55%) and other cannabinoids less than 1% in both leaf and inflorescence tissue^52^. Chemovar III showed a total CBD to THCA ratio of 1.8, which matched with its reported profile in its marketing materials. These amounts were representative of modern North American-cultivated seedless chemovars, which contain up to 25% total cannabinoids, with THCA and CBDA as the main constituents^4^. Cannabinoids mainly exist in the plant as carboxylic acids and are decarboxylated into neutral forms over time - heat or light exposure expedites decarboxylation^29^. Due to the convertibility of THCA, total THC dose is calculated as the sum of the amount of THCA multiplied by a correction factor 0.877 plus the amount of THC^29^. Neutral form cannabinoids, including CBDV, CBG, CBD, THCV, ∆^9^-THC, and CBC, were either not detected or found at several times less than acid form cannabinoids. CBN was detected at less than 0.01% in the leaf and inflorescence samples of the chemovars – this indicates that there was minimal degradation and that sample preparation was proper^52^. The ratios of total THC to total CBD matched with some of those representatives of the wide-leaflet drug (WLD) (“Indica” in the vernacular) and narrow-leaflet drug (NLD) (“Sativa” in the vernacular) biotypes^55,56^ but contradicted others^49,57^. Studies have shown that the concentrations of total THC and total CBD have no discriminatory value for chemovars in the modern vernacular (“Sativa” vs. “Indica”) due to the misuse of the botanical nomenclature, extensive cross-breeding, and unreliable labelling during unrecorded hybridization^48–51,56,58,59^.

CBDVA was detectable in Chemovar III at 0.05% but was not detected in the other two chemovars. The correlation between CBDVA and CBD is unclear, but elevated levels of CBDV and THCV are more common in *C. indica* drug biotypes (WLD and NLD) than the *C. sativa* hemp biotype^58^. CBDV is reported to rival CBD’s therapeutic potential for the treatment of epilepsy, particularly focal seizures^60^. It is also reported to have therapeutic potential for treating nausea and vomiting^61^. Total THC and total CBD ratio in the leaves of the intermediate type chemovar was consistent with that in the inflorescence, which is consistent with conclusions from other studies^62–64^. Notably, the ratio of total CBC to total THC was ten times higher in leaves than in inflorescence for all three chemovars.

### Mono- and sesquiterpenoid profile in inflorescences, leaves, stem barks, and roots

Mono- and sesquiterpenoids were not detected in stem barks or roots (Fig. 4c). Total mono- and sesquiterpenoids ranged from 0.125% to 0.278% in leaf and 1.283% to 2.141% in inflorescence in the three chemovars (Supplementary Table 12), which were less than the 4% reported in unfertilized flowers in a previous study^5^. Total sesquiterpenoid content was higher than total monoterpenoids in fan leaves in Chemovar I and Chemovar II but was comparable in Chemovar III. This observation was clearer when contents were expressed as ratios: sesquiterpenoids comprised approximately 90% of total terpenoids in Chemovar I and II and comprised 53% of the total terpenoids in Chemovar III.

The ratios of major terpenoids relative to total terpenoids in the inflorescence (Supplementary Table 13), agreed with values reported in a compiled study^29^. β-myrcene was the most abundant monoterpenoid at concentrations ranging from 16.78% to 23.57%. α-Pinene ranged between 4.26% and 36.07%. β-Pinene ranged between 3.04% and 7.12%. Limonene ranged between 3.79% and 16.42%. Linalool ranged between 2.10% and 2.99%. β-Caryophyllene was the most abundant sesquiterpenoid and ranged between 6.71% and 45.25%. α-Humulene ranged between 2.82% and 7.97%. β-Eudesmol ranged between 0.07% and 2.64%. All mono- and sesquiterpenoid ratios were consistent with previously reported essential oil contents in fresh plant material (between 47.9–92.48% and 6.84–47.5%, respectively)^29^. The ratios of individual terpenoids in the leaf were comparable to those in inflorescence for all three chemovars (Supplementary Fig. 2).

For terpenoids whose analytical standards were unavailable for sourcing, identification was performed using its mass spectrum, and semi-quantification was performed using individual response area relative to the total response area of all terpenoid peaks using GC-FID, where the response factor was taken as one^52,65–67^. Several chemotaxonomic studies utilized this method to discriminate “Sativa” and “Indica” varieties and found that terpenoid profiles are uniquely retained from their respective landrace ancestors^48–50,56,58,59^. The presence of more hydroxylated terpenoids in Chemovar III does not fit its reported classification as *C. indica* ssp. *indica* (NLD, vernacular “Sativa”), but more closely aligns with *C. indica* ssp. *afghanica* (WLD, vernacular “Indica”). Similarly, although the Chemovar I and II were reported as “Indica,” their terpenoid profiles were characteristic of “Sativa” chemovars. One study found that the reported ancestry percentages of “Sativa” vs. “Indica” for 81 drug-type chemovars are only moderately correlated with the calculated genetic structure^68^, indicating that the vernacular classifications do not reliably communicate genetic identity. For medicinal research and applications, cannabis chemovars should be identified by their chemical fingerprints, which are more reliable than their names^48,49,56^.

### Flavonoid profile in inflorescences, leaves, stem barks, and roots

A total of twenty-six flavonoids have been identified in cannabis plants, which are methylated and prenylated aglycones or conjugated *O*-glycosides or *C*-glycosides of orientin, vitexin, isovitexin, quercetin, luteolin, kaempferol, and apigenin^6,7,69^. In this study, total flavonoid content was expressed as the sum of these seven flavonoids after acid hydrolysis. Flavonoids were not detected in roots, and stem barks, less detected in the inflorescence (0.07–0.14%), and were highest in leaves (0.34–0.44%) (Fig. 4e). The total flavonoid in cannabis leaves is estimated to be around 1%^46^, which matches with our result considering flavonoids exist as both free flavonoids (aglycones) and conjugated glycosides. Flavonoid content also varied between chemovars. Total flavonoid content in inflorescence was significantly higher in Chemovar III (0.14 ± 0.002%) than Chemovar I (0.07 ± 0.001%) and Chemovar II (0.010 ± 0.005%) (n = 3, p < 0.0001) (Fig. 4f). The total flavonoid content in leaves was higher in Chemovar II (0.44 ± 0.02%) and Chemovar III (0.40 ± 0.01%) than in Chemovar I (0.34 ± 0.02%) (n = 3, p = 0.0043). Vitexin was found to be the most abundant flavonoid, ranging from 0.12% to 0.17% in leaves and 0.02% to 0.06% in the inflorescence (Fig. 4f,g, Supplementary Table 14), consistent with the previous studies^7,70^. Orientin content ranged from 0.07% to 0.08% in leaves and 0.01% to 0.03% in inflorescence in our samples, which are similar to results reported by Vanhoenacker^70^, but are lower than results reported by Flores-Sanchez and Verpoorte^7^. The analyzed isovitexin and luteolin contents were lower than other studies^7,70^. Apigenin content ranged from 0.03% to 0.07% in leaves and 0.004% to 0.01% in inflorescence in our samples, which are similar to results reported by Vanhoenacker^70^ but are lower than results reported by Flores-Sanchez and Verpoorte^7^. Neither quercetin nor kaempferol was found in leaf samples – these results are different from a previous study^7^ that reported 0.2% quercetin in leaves. The inconsistency of reported values may be caused by differences in plant age and chemovar varieties. Unlike cannabinoid accumulation, individual and total flavonoid content decreases as the plants age^7^. Orientin, vitexin, and their glucosides were reported to have value in discriminating cannabis subspecies^69^. Cannflavin A and B are also notable flavonoids with medicinal potential identified in cannabis^71^. However, due to the unavailability of reference standards at the time, they were not included in this study.

### Sterol profile in inflorescences, leaves, stem barks, and roots

Total sterol content was expressed as the sum of campesterol, stigmasterol, and β-sitosterol, increased from inflorescences, leaves, roots, to stem barks (Fig. 4h). The ratio of three sterols was consistent with a previous study on cannabis roots^72^. β-sitosterol was the most abundant sterol in roots and stem barks for all three chemovars, ranging from 0.04 to 0.06% (Fig. 4i,j, Supplementary Table 15). Campesterol content ranged between 0.01% to 0.02% in roots and stem barks and was not detected in leaf. Stigmasterol had the lowest concentration in roots and stem barks at 0.01% and was most concentrated in leaves at 0.03%. Total sterols in stem barks were comparable between three chemovars (n = 3, p = 0.0550) while they were significantly different in root material (n = 3, p < 0.0001). Campesterol was not significantly different in the stem barks of three chemovars (n = 3, p = 0.3523) but was significantly different (n = 3, p < 0.0001) in root material. Stigmasterol was significantly different in stem barks in three chemovars (n = 3, p = 0.0012) and in root material (n = 3, p < 0.0001). β-sitosterol was significantly different in the roots of three chemovars (n = 3, p < 0.0001) but less variable in the stem barks of three chemovars (n = 3, p = 0.1216).

### Triterpenoids profile inflorescences, leaves, stem barks, and roots

Total triterpenoid content was expressed as the sum of β-amyrin, epifriedelanol, and friedelin. It increased from inflorescences (not detected), to leaves (<0.05%), stem barks (0.05–0.15%), and roots (0.1–0.3%) (Fig. 4k). Total triterpenoid in both the roots and stem barks in Chemovar III was significantly higher than in Chemovar I and II (n = 3, p < 0.0001). Friedelin is the most prominent triterpenoid in cannabis and is concentrated in the stem barks and roots^5^ (Fig. 4l,m). It ranged from 0.083% to 0.135% in roots and 0.033% to 0.100% in stem barks (Supplementary Table 16). The results were significantly higher than the 0.00128% (12.8 mg/kg) reported in a previous study^72^. Epifriedelanol was found to range from 0.033% to 0.092% in roots and 0.013% to 0.041% in stem barks, which was higher than the 0.00213% (21.3 mg/kg) previously reported^72^. Chemovar III had significantly higher friedelin, epifriedelanol, and β-amyrin in stem barks and roots than the other chemovars (n = 3, p < 0.0001). Neither friedelin nor epifriedelanol was found in leaf samples. Conversely, β-amyrin was found to be higher in leaf (0.012% to 0.026%) than in stem bark (0.006% to 0.007%) or root (0.005% to 0.013%).

## Discussion

To bridge traditional medicine and modern evidence-based medicine, biochemically active compounds must be identified, and their molecular mechanisms determined through preclinical and clinical studies. The secondary metabolites quantified in each part of cannabis are summarized in Supplementary Table 17. Since concentrations above 0.05% are pharmacologically interesting^38^, cannabis inflorescence and leaf material may contain sufficient cannabinoids, mono- and sesquiterpenoids, and flavonoids for therapeutic applications. For example, the leaves of Chemovar III contain 0.34% flavonoids in terms of total aglycones. In comparison, ginkgo leaves, which are used for ginkgo extract and are among the best sources of flavonoids, contains 0.4% total flavonoids in terms of total aglycones^73^. The stem bark and root are sources of triterpenoids and sterols. For example, friedelin is found in the leaves of *Azima tetracantha* Lam. (bee sting bush), containing a relatively high amount at 0.36%^74^. In comparison, dried cannabis roots and stem barks contain between 0.1% to 0.15%. Friedelin is also isolated from the dried leaves of *shorea robusta* (shala tree), which has been commonly used in traditional Indian medicine^75^. Cannabis contains more than ten times more friedelin than shala tree. The potential therapeutic properties of the identified compounds have been comprehensively reviewed^5,76^. For example, terpenoids and flavonoids identified in inflorescences and leaves have anti-inflammatory, anti-rheumatic, analgesic, anticonvulsant, antioxidant and neuroprotective, larvicidal, gastroprotective properties, and beneficial effects on the respiratory system^77–80^. Triterpenoids and sterols identified in stem barks and roots have anti-inflammatory, analgesic, antimicrobial, antioxidant, neuroprotective, angiogenic, anti-osteoarthritic, and estrogenic properties^81^. These secondary metabolites possess pharmaceutical values and may contribute to the overall therapeutic benefits of cannabis; however, these synergies require further investigation to provide direct evidence. Such evidence should be derived from studies using cannabis material instead of proxy studies of extracts from other plants.

In history, cannabis has been indicated for a wide range of conditions relating to pain, inflammation, and mental illness. For example, the inflorescences were used in traditional Chinese medicine for conditions including acute pain, mania, insomnia, coughing/panting, and wounds. The leaves were indicated for malaria, panting, roundworm, scorpion stings, hair loss, greying of hair. The stem barks were used for strangury and physical injury. The roots were used for strangury, spotting, vaginal discharge, difficult births, retention of the placenta, and physical injury^32,33^. Although the terminology in historical texts may be different from modern science and the nuances lost in the translation between Chinese and English, the uses of cannabis inflorescence indicated in ancient Chinese literature are comparable to those found in modern preclinical and clinical studies for cannabinoids^76,82–86^. But modern medicine has not fully developed the medical potential of cannabis leaf, stem bark, and root. Their traditional use may be used as a point of reference for clinical research. Similarly, the study of biomechanisms and the clinical effects of individual compounds may be consolidated for the development of applications using each plant part. For example, ∆^9^-THC has antiemetic and appetite stimulant properties and have been used to treat nausea or vomiting associated with chemotherapy and anorexia associated with AIDS-related weight loss by two approved medicine Marinol (dronabinol, synthetic ∆^9^-THC) and Cesamet (nabilone, a THC-derivative)^86^. In addition, other previously unapplied cannabinoids have also been proven to have antiemetic and appetite stimulant properties, including CBDV^87^, CBD^88^, CBDA^89–93^, and THCA^94^, and may improve the therapeutic potential and reduce undesired side effects when used synergistically with other compounds in the plant material. Modern research also indicates that cannabis and cannabinoids have therapeutic potential for multiple sclerosis, Huntington’s disease, Parkinson’s disease, glaucoma, hypertension, stress and psychiatric disorders, Alzheimer’s disease and dementia, and anti-neoplasia, many of which have not been described in traditional use. Identification of bioactive compounds followed by well-designed clinical studies can convert each part of cannabis plant into evidence-based medicine.

## Conclusions

Secondary metabolites, including cannabinoids, terpenoids, sterols, and flavonoids, were individually profiled in cannabis inflorescences, leaves, stem barks, and roots for three chemovars. Inflorescences and leaves are relatively abundant in cannabinoids, monoterpenoids, sesquiterpenoids, and flavonoids. Stem barks and roots contain triterpenoids and sterols. These bioactive compounds may underlie the traditional medicinal applications of each cannabis plant part in various cultures over thousands of years of cultivation. A comprehensive profile of bioactive compounds and thorough investigations of their synergistic interactions enables the correlation between plant compositions and therapeutic effects, ultimately bridging traditional herbal medicine with modern science. This approach enables the development of new cannabis-based medicine using all or subsets of plant parts, as opposed to the inflorescence only. One future trend for the cannabis industry is to fully utilize each part of cannabis by applying modern scientific methodologies for validating its traditional use.

## Materials and Methods

### Solvents and chemicals

The 14 cannabinoid standards and ∆^9^-THC-d_3_, which was used as an internal standard (IS), were purchased from Sigma-Aldrich Company (Oakville, ON, Canada). All cannabinoid standards were analytical grade 1 mg/mL solution in methanol or acetonitrile. Standards for monoterpenoids (α-pinene, camphene, β-pinene, myrcene, Δ^3^-carene, α-terpinene, p-cymene, limonene, β-ocimene, γ-terpinene, terpinolene, linalool, 1,8-cineole (eucalyptol), (−)-isopulegol, geraniol) and sesquiterpenoids (β-caryophyllene, α-humulene, trans-nerolidol, (−)-guaiol, α-bisabolol, and caryophyllene oxide) were purchased from RESTEK (Bellefonte, PA, US). These 21 terpenoids were certified reference materials provided as mixed standards at approximately 2500 µg/mL in isopropanol. Standards for monoterpenoids (α-phellandrene, sabinene hydrate, camphor, fenchol, borneol, α-terpineol, sabinene, (+)-carvone, (+)-dihydrocarvone, pulegone, terpineol-4-ol, fenchone, and geranyl acetate), sesquiterpenoids (aromadendrene, (+)-cedrol, globulol, ledene, viridiflorol), triterpenoid (friedelin), sterol (stigmasterol), flavonoids (orientin, vitexin, isovitexin), tridecane (used as an IS for quantification of mono- and sesquiterpenoids), and cholesterol (used as an IS for quantification of triterpenoids and sterols) were analytical standards purchased from Sigma-Aldrich Company (Oakville, Ontario, Canada). Sesquiterpenoids (trans-β-farnesene and valencene) were certified reference materials purchased from ChromaDex (Irvine, CA, US). Standards for nerol, sesquiterpenoids (β-eudesmol and β-elemene), triterpenenoids (β-amyrin and epifriedelanol), sterols (campesterol and β-sitosterol), and flavonoids (quercetin, luteolin, kaempferol, and apigenin) were certified reference materials purchased from Chengdu Push Bio-Technology Co., Ltd. (Chengdu, Sichuan, China). Methanol, ethanol, hexane, and hydrochloric acid were purchased from Fisher Scientific (Ottawa, ON, Canada). Ethyl acetate and formic acid were purchased from Caledon Laboratory Chemicals (Halton Hills, Ontario, Canada). C7–C40 saturated alkanes standard was purchased from Sigma-Aldrich Company (Oakville, Ontario, Canada). Water was produced in-house using a Millipore filtration system, which purified water to 18mΩ resistivity.

### Sample collection and preparation

Dried cannabis inflorescence and fresh leaves, stems, and roots from three cannabis chemovars were provided by a licensed producer in Canada (Supplementary Fig. 1). The plants were kept vegetative for six months and flowered for two months in a greenhouse. Stems were taken near the root tissue. Stems from the upper parts of the plants were not available. Two THC dominant chemovars (Chemovar I - Grand Doggy Purps and Chemovar II - Granddaddy Purple) were alleged to be “Indica” varieties. The intermediate type chemovar (Chemovar III - CBD Mango Haze), characterized by having a total THC to total CBD ratio of 1:2, was purported to be a “Sativa” variety. Five to eight flower heads (2 g to 4 g) of each chemovar were pulverized with a manual grinder. Stem barks and roots, which were cut into 2 cm pieces, were air-dried together with leaves at room temperature for 24 hours. Dried leaf material was crushed using a mortar and pestle and sifted through a 1.18 mm sieve. Dried stem bark and root samples were ground into a fine powder using an electric blender. All dried material was stored under refrigeration for two weeks until analysis.

### Methanol extraction for cannabinoids, monoterpenoids, and sesquiterpenoids

In brief, 400 mg plant material was extracted with 20.0 mL methanol (with 100 µg/mL tridecane as IS for mono-and sesquiterpenoids) by sonication for 20 minutes at room temperature. The extract was then filtered through a 0.45 µm membrane filter disk. An aliquot of the extract was used to quantify mono- and sesquiterpenoids using GC-MS. For cannabinoids, the prepared solutions were spiked with ∆^9^-THC-d_3_ (0.5 μg/mL) as IS prior to LC-MS analysis. Dilutions were applied as necessary.

### Ethyl acetate extraction for triterpenoids and sterols

One gram of dried sample was extracted with 20.0 mL ethyl acetate by sonication for one hour, followed by maceration for one day at room temperature. The extract was filtered through a 0.45 μm membrane filter disk and spiked with cholesterol (50 μg/mL) as IS prior to GC-MS analysis.

### Acid-hydrolyzation for flavonoids

The method for acid hydrolysis extraction of flavonoids was adapted from the monograph for ginkgo in the latest version of the United States Pharmacopoeia (USP)^95^. In brief, 250 mg of the sample was extracted with 5 mL extraction solvent (ethanol, water, and hydrochloric acid at a 50:20:8 volume ratio). The air in the tube was displaced with nitrogen. The solution was then vortexed for 10 seconds and sonicated for 10 minutes, followed by hydrolysis in a 100 °C water bath for 135 minutes. The tube was left to cool to room temperature. Then the contents were transferred to a 50 mL volumetric flask. The tube was then repeatedly rinsed with methanol, and the rinses were combined with the extract. The flask was filled to volume with methanol, then sonicated again for 5 minutes. The solution was filtered through a 0.45μm membrane filter disk, an aliquot of which was used for quantification.

### LC-ESI-MS setup for cannabinoids assay

The LC-ESI-MS system used in this study was a modular Agilent 1260 Infinity II LC system comprised of the following components: a vacuum degasser, a quaternary pump (G7111B), an autosampler (G7129A), an integrated column compartment (G7130-60030), and a single quadrupole liquid chromatography/mass selective detector (LC/MSD 6125B) with electrospray ionization (ESI) (C1960-64217). The chromatographic separation of cannabinoids was performed on an Agilent Zorbax RX-C18 column (4.6 mm × 150 mm, 3.5 μm). The mobile phase was composed of 0.2% aqueous formic acid (A) and methanol (B). Gradient elution was as follows: 75–90% B in 0–13 minutes and 90% B in 13–26 minutes. The post-run time was 4 minutes. The flow rate was 0.6 mL/min. The column temperature was set at 30 °C. The injection volume was 5 µL. The ESI-MS system was operated in positive ionization mode. Mass to charge ratios (M/z) of fragment ions for each compound were listed in Supplementary Table 18. The instrument settings were set as follows: the capillary voltage was 3 kV, the nebulizer (N_2_) pressure was 50 psi, the drying gas temperature was 350 °C, the drying gas flow was 12 L/min, and the fragmentor voltage was 70 V.

### HPLC-UV-MS setup for flavonoid identification and quantification

The HPLC-UV-MS system used in this study was the same LC-MS system described above, with an Agilent 1260 variable wavelength detector (G7114A) in series. The chromatographic separation of flavonoids was performed on Phenomenex Synergi polar-RP 80 Å LC column (4.6 mm ×150 mm, 4 μm). The mobile phase was composed of 0.2% aqueous formic acid (A) and methanol (B). Gradient elution was as follows: 30% B in 0–3 minutes and 30–60% in 3–50 minutes. The post-run time was 5 minutes. The flow rate was 1.0 mL/min. The column temperature was set at 30 °C. The injection volume was 5 µL. The ESI-MS system was operated in negative ionization mode. M/z for of fragment ions for each compound were listed in Supplementary Table 19. The instrument settings were set as follows: the capillary voltage was 3 kV, the nebulizer (N_2_) pressure was 50 psi, the drying gas temperature was 350 °C, the drying gas flow was 10 L/min, and the fragmentor voltage was 70 V. The UV detector was monitored at 350 nm for quantification of seven flavonoids.

### GC-MS setup for terpenoids and sterols assay

The GC-MS system used in this study was an Agilent 7890 A GC system comprised of the following components: an Agilent 7890 A Gas Chromatograph (G3440A), an Agilent 5975 C inert MSD with triple-axis detector, a K′(Prime) GC sample injector (MXY 02-01B), and a Phenomenex ZB-5MSi column (30 m × 0.25 mm, 0.25 µm). A temperature gradient program was used for the separation of mono- and sesquiterpenoids: 40 °C for 2 minutes, ramp of 20 °C/min up to 100 °C, ramp of 5 °C/min up to 160 °C, and ramp of 20 °C/min up to 280 °C. Run time was 20 minutes. The injector temperature was 280 °C. Injection volume was 1 µL. Split ratio was 10:1. The carrier gas (helium) flow rate was 1.2 mL/min. The MS source was set to 230 °C, the single quad temperature was 150 °C, and the transfer line temperature was set to 280 °C. The mass spectrometer was operated in selected ion monitoring (SIM) mode. Quantifier and qualifier ions for each compound were listed in Supplementary Table 3. A second temperature gradient program was used for the quantification of triterpenoids and sterols: 80 °C for 1 minute, the ramp of 20 °C/min up to 250 °C, and ramp of 10 °C/min up to 300 °C. Run time was 34.5 minutes. The injector temperature was 300 °C. The injection volume was 1 µL and splitless. The carrier gas (helium) flow rate was 1.5 mL/min. The MS source was set to 230 °C, the single quad temperature was 150 °C, and the transfer line temperature was set to 280 °C. The mass spectrometer was operated in SIM mode. Quantifier and qualifier ions for each compound were listed in Supplementary Table 20–21.

### GC-FID setup for terpenoids identification and semi-quantification

Terpenoids without available standards were identified and semi-quantified using GC-MS for identification and GC-FID for semi-quantification, respectively. A Hewlett Packard 5890 Series II GC equipped with a 7673 A automatic injector and a flame ionization detector (FID) was used for the analysis of the available terpenoids standards and samples. The instrument was equipped with a ZB-5HT capillary column (30 m × 0.25 mm ID, 0.25 µm film thickness) with the injector temperature at 280 °C, an injection volume of 2 µL, a split ratio of 10:1, and carrier gas (helium) flow rate of 1.2 mL/min. The temperature gradient started at 60 °C and increased at a rate of 3 °C/min until 280 °C. The total run time was 75 minutes. The mass scan range was from 30 amu to 550 amu. One 1000 μg/mL saturated alkanes standard (C7–C40), one 100 μg/mL mixed terpenoid standard, and the samples were injected using the temperature gradient program above. The linear retention index (LRI) was calculated by comparing the retention time of one terpenoid compound (t_R,i_) with those of n-alkanes with n carbons eluted before the compound (t_R,n_) and with n + 1 carbons eluted after the compound (t_R,n+1_)^96^:

$${\rm{LRI}}\,({\rm{target}}\,{\rm{compound}})=100\times (\frac{{t}_{R,i}-{t}_{R,n}}{{t}_{R,n+1}-{t}_{R,n}}+n)$$

Each LRI was compared to the data listed in the NIST Chemistry WebBook^97^. The mass spectrum of the target compound was compared to data in the NIST mass-spectra database embedded in the GC-MS system. If both the LRI and the mass spectrum confirm the identity of the target compound, then the compound can be semi-quantified by comparing the response area of the target compound and a closely eluted compound with known concentration while assuming that the relative response factor is one^52,67^.

### Sample preparation optimization

Sample preparation procedures were compared and optimized step by step. Two pulverization methods, which were manual grinding and electric blending, were compared for preparing cannabis inflorescence material. The extraction efficiency of solvents (methanol vs. methanol/chloroform (9/1, v/v))^29,98^ for cannabinoids were compared. Extraction durations (sonication for 10 vs. 20 vs. 30 minutes vs. maceration for one day) were studied using the yield of total extracted cannabinoids and total extracted mono- and sesquiterpenoids. The effect of sonication temperature (20 °C vs. 30 °C vs. 50 °C) and the number of extractions (once vs. twice vs. thrice) were also compared for cannabinoids. For triterpenoids and sterols, the compared extraction methods were sonication for 1 hour and maceration for 1, 2, 3, 4, or 5 days in terms of total triterpenoids and total sterols extracted. Five duplicate samples were tested for each scenario. To investigate potential interference from cannabinoids during flavonoids testing, the effects of the hexane wash before acid hydrolysis were examined by comparing flavonoids yields.

### Method validation

Developed methods were validated for selectivity, linearity, trueness, precision (repeatability and intermediate precision), LOD, LOQ, and robustness (using a different column, instrument, and analysts). Measurement uncertainty (accuracy) was determined using the total error concept^99^. Matrix effects and extraction efficiency were also determined for cannabinoids.

### Selectivity

Selectivity was determined by injecting a solvent blank to confirm that there were no false signal peaks at the targeted retention time. Each compound standard was individually injected to determine retention times for GC and LC analysis. Representative chromatograms were used to demonstrate selectivity. Each compound was labelled correspondingly.

### Calibration curve and linearity

A 100 µg/mL mixed standard solution of 14 cannabinoids was further diluted to 1, 0.5, 0.1, 0.05, and 0.01 µg/mL to construct a linear regression curve. The linearity of the responses was confirmed visually by plotting residuals against concentrations. Similarly, calibration curves for mono- and sesquiterpenoids were constructed at concentrations of 1, 5, 25, 50, 100, and 250 µg/mL. Calibration curves for triterpenoids and sterols were constructed at concentrations of 1, 5, 25, 50, and 100 µg/mL. For flavonoids, a 100 μg/mL mixed standard was further diluted into 25, 10, 5, 2, and 1 μg/mL to construct calibration curves.

### LOD and LOQ

Multiple duplicate standards that had concentrations near the expected LOD were measured^100^. The LOD is the minimum concentration that distinguishable from method blank results within 99% confidence. The relative standard deviations (RSD) of integrated areas were used for LOD determination as follows:

$$\mathrm{LOD}\,(\mu {\rm{g}}/\mathrm{mL})={{\rm{t}}}_{{\rm{\alpha }}}\times \mathrm{RSD}\times \mathrm{the}\,\mathrm{concentration}\,\mathrm{of}\,\mathrm{injected}\,\mathrm{standard}\,(\mu {\rm{g}}/\mathrm{mL})$$

where t_α_ is the confidence factor from the Student’s t-distribution table (one-sided), and α is the significance level (α = 0.01). For ten injections, t_α_ = 2.821. LOQ is three times LOD. The repeatedly injected concentration was 0.01 µg/mL for cannabinoids, 0.5 µg/mL for terpenoids and sterols, and 1 µg/mL for flavonoids.

### Trueness, precision, and accuracy

Accuracy, expressed as the total error of a method, is affected by systematic error (trueness) and random error (precision). This study determined accuracy using the total error concept^101^. Low, medium and high concentrations of mixed standards were spiked into blank matrices and tested for trueness and precision. Mint leaves were prepared as the cannabinoid blank matrix. For terpenoids and sterols, cannabis plant material was repeatedly washed using organic solvent until the measured terpenoids and sterols levels were below LOD. Approximately 200 mg of the blank matrices were spiked with three levels (0.1, 0.5, and 1.0 μg/mL for cannabinoids, 50, 100, and 200 µg/mL for mono- and sesquiterpenoids, and 5, 10, and 25 µg/mL for triterpenoids and sterols) of mixed standards with three replicates for each concentration level analyzed for each of three consecutive days. The trueness was calculated using the following equation:

$$\begin{array}{ccc}{\rm{Relative}}\,{\rm{bias}}\,( \% ) & = & \frac{{\rm{Measured}}\,{\rm{spiked}}\,{\rm{value}}-{\rm{Nominal}}\,{\rm{spiked}}\,{\rm{value}}}{{\rm{Nominal}}\,{\rm{spiked}}\,{\rm{value}}}\times 100 \% \\  & = & {\rm{Recovery}}( \% )-100 \% \end{array}$$

The measured values were averaged to calculate relative bias for each level (n = 9). Cannabis leaves were used to spike three levels of flavonoid standards (20, 50, and 80 µg for orientin and isovitexin; 20, 30, and 80 μg for vitexin; 50, 100, and 150 µg for quercetin, luteolin, kaempferol, and apigenin) for three replicates before hydrolysis.

### Repeatability and intermediate precision

The intraday repeatability was determined as the RSD from assaying blank matrix samples spiked with three levels of standards. Repeatability was calculated as the pooled RSD over the three intraday RSD values:

$${{\rm{RSD}}}_{{\rm{pooled}}}=\sqrt{\frac{{{\rm{RSD}}}_{1}^{2}+{{\rm{RSD}}}_{2}^{2}+{{\rm{RSD}}}_{3}^{2}}{3}}$$

Intermediate precision for all analytes except flavonoids was evaluated as the RSD at each concentration level for the average measured value of nine replicates over three days. Intermediate precision for flavonoids was calculated as RSD for twelve replicates by testing six samples for two consecutive days by two analysts.

### Accuracy

The total uncertainty in measurement combines bias and error in intermediate precision, and is calculated as follows:

$${\rm{M}}{\rm{e}}{\rm{a}}{\rm{s}}{\rm{u}}{\rm{r}}{\rm{e}}{\rm{m}}{\rm{e}}{\rm{n}}{\rm{t}}\,{\rm{u}}{\rm{n}}{\rm{c}}{\rm{e}}{\rm{r}}{\rm{t}}{\rm{a}}{\rm{i}}{\rm{n}}{\rm{t}}{\rm{y}}\,({\rm{t}}{\rm{o}}{\rm{t}}{\rm{a}}{\rm{l}}\,{\rm{e}}{\rm{r}}{\rm{r}}{\rm{o}}{\rm{r}})=\sqrt{{({\rm{r}}{\rm{e}}{\rm{l}}{\rm{a}}{\rm{t}}{\rm{i}}{\rm{v}}{\rm{e}}{\rm{b}}{\rm{i}}{\rm{a}}{\rm{s}})}^{2}+{({\rm{R}}{\rm{S}}{\rm{D}}({\rm{i}}{\rm{n}}{\rm{t}}{\rm{e}}{\rm{r}}{\rm{m}}{\rm{e}}{\rm{d}}{\rm{i}}{\rm{a}}{\rm{t}}{\rm{e}}{\rm{p}}{\rm{r}}{\rm{e}}{\rm{c}}{\rm{i}}{\rm{s}}{\rm{i}}{\rm{o}}{\rm{n}}))}^{2}}$$

### Matrix effect and extraction efficiency

The matrix effect for cannabinoids was measured by comparing the response of the blank matrix extract spiked with three levels of standards (low, medium, high) to the response of standards of the same concentration in the solvent, calculated as:

$${\rm{Matrix}}\,{\rm{effect}}=\frac{{\rm{Analyte}}\,{\rm{response}}\,({\rm{post}} \mbox{-} {\rm{extraction}}\,{\rm{spiked}}\,{\rm{blank}}\,{\rm{matrix}})}{{\rm{Analyte}}\,{\rm{response}}\,({\rm{solvent}})}\times 100 \% $$

Extraction recovery is measured by comparing the response of the blank matrix before and after extraction, calculated as:

$${\rm{Extraction}}\,{\rm{efficiency}}=\,\frac{{\rm{Analyte}}\,{\rm{response}}\,({\rm{pre}} \mbox{-} {\rm{extraction}}\,{\rm{spiked}}\,{\rm{blank}}\,{\rm{matrix}})}{{\rm{Analyte}}\,{\rm{response}}\,({\rm{post}} \mbox{-} {\rm{extraction}}\,{\rm{spiked}}\,{\rm{blank}}\,{\rm{matrix}})}\times 100 \% $$

### Robustness

The robustness of the cannabinoids LC-MS method was verified using an alternate analytical column and a different LC-MS instrument of the same model. The robustness of the GC-MS method for mono- and sesquiterpenoids was verified by an alternate analyst who quantified the same sample batch on an alternate day. Six duplicate samples were tested for each scenario.

### Statistical analysis

Each experiment was independently repeated five times for sample preparation optimization. Each compound was measured three times for each plant part. Data are expressed as mean ± standard deviation (SD). Data sets were compared using the two-sided Student’s *t*-tests at the 0.05 significance level. For multiple groups, one-way ANOVA followed by Tukey honestly significant difference (HSD) post hoc test at the 0.05 significance level were used.

## Supplementary information

Supplementary materials.

## Data Availability

All relevant data are available from the authors upon request.
