# Supplementary material for: Secondary Metabolites Profiled in Cannabis Inflorescences, Leaves, Stem Barks, and Roots for Medicinal Purposes
Source: Sci Rep. 2020 Feb 24;10:3309. doi: 10.1038/s41598-020-60172-6 (PMC7039888; doi:10.1038/s41598-020-60172-6)
Supplement: Supplementary file 1 — Supplementary materials. [file 41598_2020_60172_MOESM1_ESM.pdf]

## Supplementary information of

# Secondary Metabolites Profiled in Cannabis Inflorescences, Leaves, Stem Barks, and Roots for Medicinal Purposes

Dan Jin[1,2], Kaiping Dai[2], Zhen Xie[2], and Jie Chen*[,1,3]

[1]Biomedical Engineering Department, University of Alberta, Edmonton, Alberta, Canada

[2]Labs-Mart Inc., Edmonton, Alberta, Canada

[3]Electrical and Computer Engineering Department, University of Alberta, Edmonton, Alberta, Canada

*Corresponding author email jc65@ualberta.ca

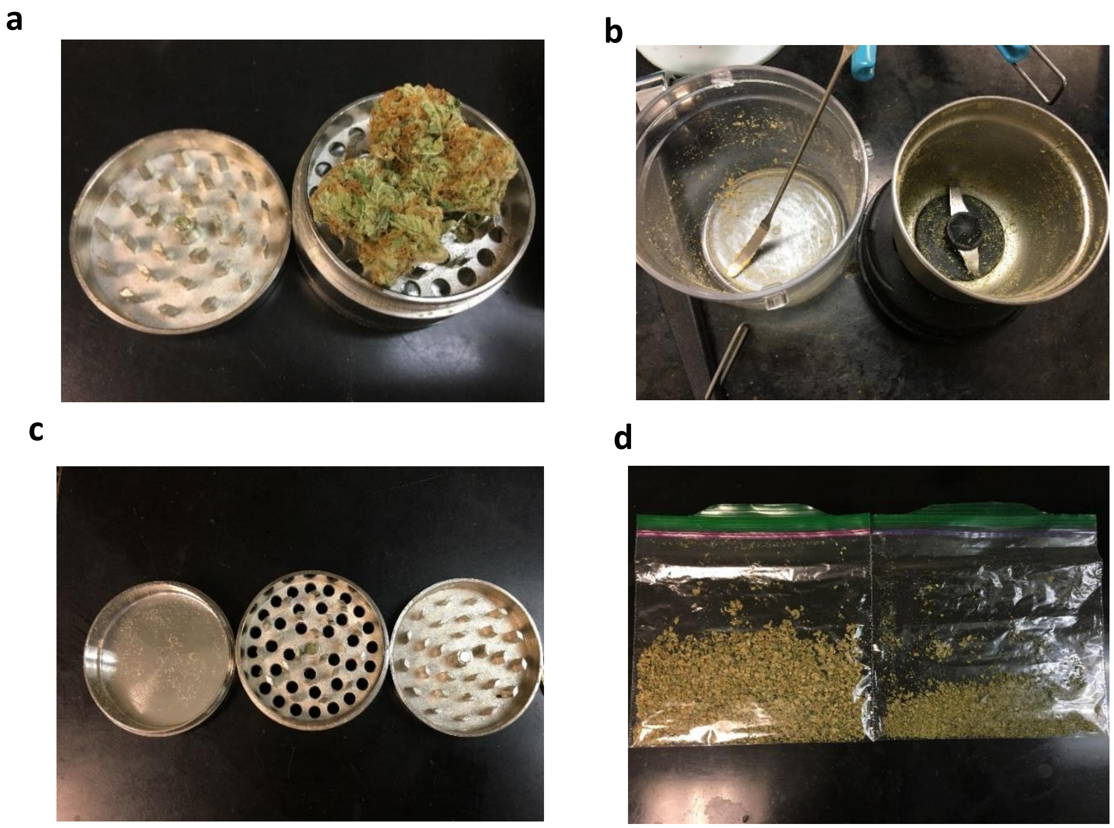

**Supplementary Figure 1.** Pulverization of cannabis raw samples using **a.** manual grinder and **b.** electric blender. **c.** The inside of manual grinder is relatively clean without much resin left behind whereas there is a layer of resin stuck to the inside of the cup of the electric blender. **d.** The resultant particle size is larger using the manual grinder (left) than the electric blender (right).

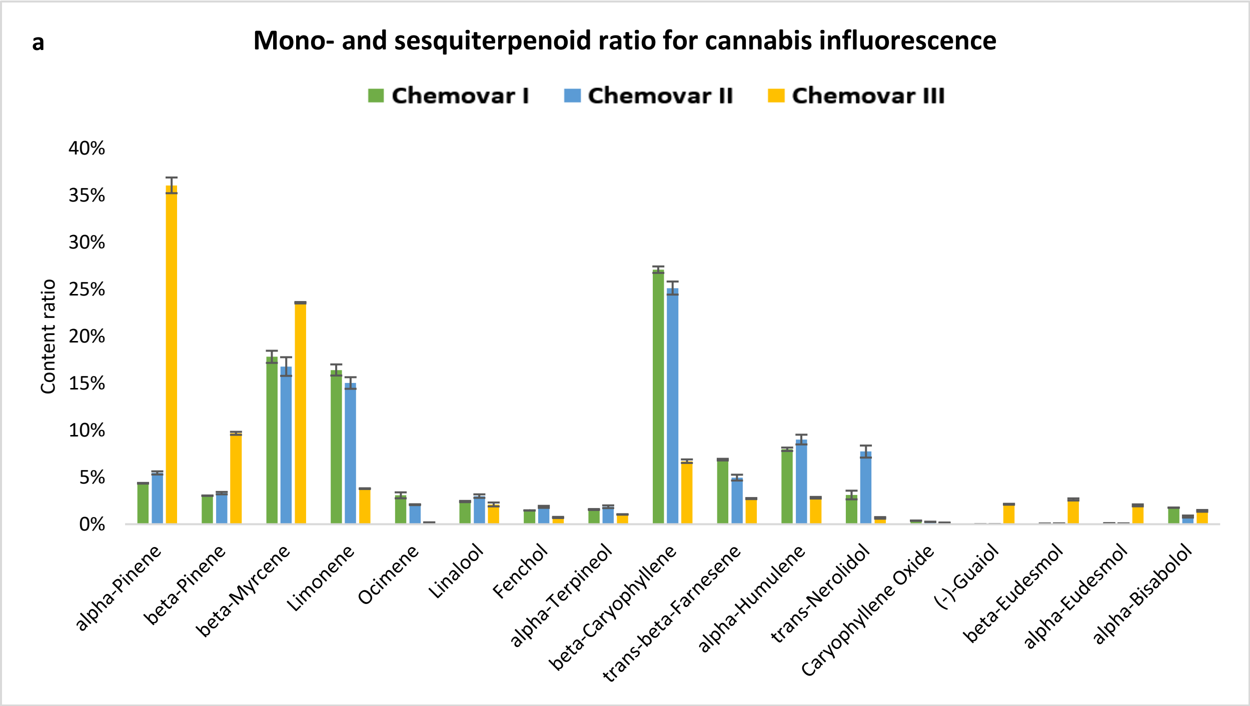


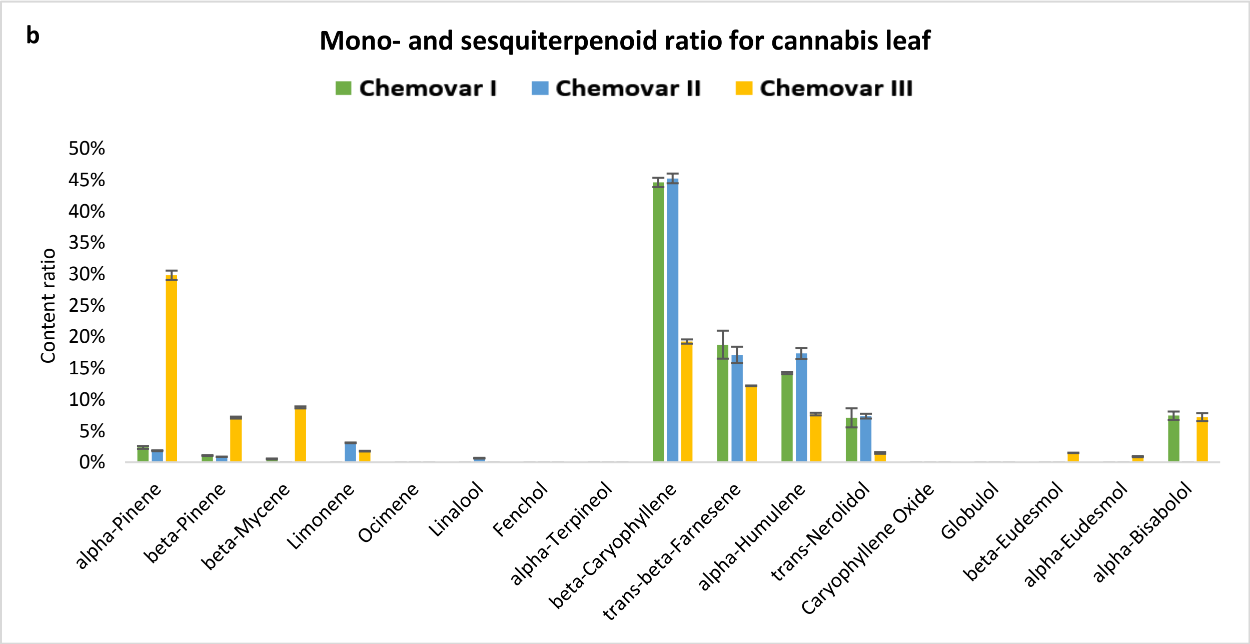


**Supplementary Figure 2. Mono- and sesquiterpenoid ratios in inflorescences and leaves in three strains. a.** Mono- and sesquiterpenoid ratios in inflorescence. **b.** Mono- and sesquiterpenoid ratios in leaves. The absolute values and ratios of individual terpenoids were consistent when compared among three strains, although total terpenoid content was significantly different.

**Supplementary Table 1. ANOVA regression statistics for cannabinoids.**

| | Quantification Range (µg/mL) | Correlation Coefficient $R^2$ | Slope ± SD | LOD (µg/mL) | LOQ (µg/mL) |
|---|---|---|---|---|---|
| 1. CBDV | 0.01-1.00 | 1.0000 | 1.3246±0.0031 | 0.001 | 0.003 |
| 2. CBDVA | 0.01-1.00 | 1.0000 | 0.7432±0.0008 | 0.002 | 0.005 |
| 3. CBG | 0.01-1.00 | 0.9998 | 0.8367±0.0053 | 0.002 | 0.005 |
| 4. CBD | 0.01-1.00 | 1.0000 | 1.3841±0.0040 | 0.0005 | 0.001 |
| 5. CBDA | 0.01-1.00 | 1.0000 | 0.7174±0.0004 | 0.001 | 0.003 |
| 6. THCV | 0.01-1.00 | 1.0000 | 1.8763±0.0022 | 0.0004 | 0.001 |
| 7. CBGA | 0.01-1.00 | 1.0000 | 0.6690±0.0015 | 0.003 | 0.008 |
| 8. CBN | 0.01-1.00 | 0.9999 | 2.9331±0.0127 | 0.001 | 0.002 |
| 9. $\Delta^9$-THC | 0.01-1.00 | 1.0000 | 1.8042±0.0024 | 0.001 | 0.003 |
| 10. $\Delta^8$-THC | 0.01-1.00 | 1.0000 | 0.9145±0.0009 | 0.002 | 0.005 |
| 11. THCVA | 0.01-1.00 | 0.9999 | 0.5474±0.0028 | 0.003 | 0.010 |
| 12. CBC | 0.01-1.00 | 1.0000 | 0.6329±0.0018 | 0.002 | 0.007 |
| 13.THCA | 0.01-1.00 | 1.0000 | 0.5700±0.0012 | 0.003 | 0.009 |
| 14. CBCA | 0.01-1.00 | 0.9998 | 0.2333±0.0017 | 0.004 | 0.012 |

**Supplementary Table 2**. Trueness, precision, and accuracy for cannabinoids

| Analyte | Spike 0.1 µg/mL | | | | Spike 0.5 µg/mL | | | | Spike 1.0 µg/mL | | | |
|---|---|---|---|---|---|---|---|---|---|---|---|---|
| | Repeatability - Pooled RSD% (n=3) | Intermediate precision - RSD% (n=9) | Trueness - Relative bias% (n=9) | Accuracy - Uncertainty of measurement% (n=9) | Repeatability - Pooled RSD% (n=3) | Intermediate precision - RSD% (n=9) | Trueness - Relative bias% (n=9) | Accuracy - Uncertainty of measurement% (n=9) | Repeatability - Pooled RSD% (n=3) | Intermediate precision - RSD% (n=9) | Trueness - Relative bias% (n=9) | Accuracy - Uncertainty of measurement% (n=9) |
| **1. CBDV** | 1.0 | 1.7 | -4.0 | 4.3 | 1.0 | 4.8 | -0.1 | 4.8 | 0.9 | 3.6 | -2.6 | 4.4 |
| **2. CBDVA** | 1.7 | 1.5 | 0.2 | 1.5 | 0.8 | 4.6 | -0.3 | 4.6 | 1.2 | 3.8 | -2.7 | 4.7 |
| **3. CBG** | 3.0 | 12.3 | 0.9 | 12.3 | 6.5 | 6.1 | 2.1 | 6.4 | 4.0 | 7.7 | -5.2 | 9.3 |
| **4. CBD** | 1.7 | 2.0 | -4.8 | 5.2 | 0.7 | 4.9 | -1.1 | 5.1 | 1.0 | 3.9 | -3.1 | 5.0 |
| **5. CBDA** | 1.7 | 3.7 | -4.2 | 5.6 | 1.3 | 4.7 | -1.2 | 4.9 | 1.3 | 4.2 | -3.4 | 5.4 |
| **6. THCV** | 1.5 | 2.3 | -4.1 | 4.7 | 0.6 | 4.8 | -1.0 | 4.9 | 1.0 | 4.0 | -3.0 | 5.0 |
| **7. CBGA** | 3.8 | 3.5 | -1.8 | 3.9 | 0.9 | 4.7 | -1.2 | 4.9 | 0.9 | 3.7 | -3.5 | 5.1 |
| **8. CBN** | 0.9 | 2.6 | -6.4 | 6.9 | 0.6 | 5.5 | -2.5 | 6.1 | 1.1 | 4.6 | -4.2 | 6.2 |
| **9. $\Delta^9$-THC** | 1.9 | 2.6 | -3.9 | 4.7 | 0.5 | 5.3 | -1.3 | 5.5 | 1.3 | 4.2 | -3.3 | 5.3 |
| **10. $\Delta^8$-THC** | 1.7 | 3.7 | -4.8 | 6.0 | 0.4 | 5.4 | -1.4 | 5.6 | 1.0 | 4.4 | -3.9 | 5.9 |
| **11. THCVA** | 3.2 | 4.0 | -4.8 | 6.2 | 0.7 | 4.7 | -1.4 | 4.9 | 1.4 | 4.4 | -4.0 | 5.9 |
| **12. CBC** | 1.9 | 3.7 | -6.3 | 7.3 | 0.7 | 5.0 | -1.2 | 5.2 | 1.1 | 4.7 | -3.5 | 5.9 |
| **13.THCA** | 9.2 | 12.0 | 0.1 | 12.0 | 6.4 | 5.5 | -1.2 | 5.6 | 1.2 | 5.0 | -4.2 | 6.6 |
| **14. CBCA** | 8.5 | 8.8 | 6.9 | 11.2 | 1.8 | 4.7 | 0.0 | 4.7 | 0.9 | 3.7 | -3.2 | 4.8 |

.

**Supplementary Table 3. Matrix effect and extraction efficiency (recovery).**

| Analyte | Spike 0.1µg/mL | | Spike 0.5µg/mL | | Spike 1.0 µg/mL | |
|---|---|---|---|---|---|---|
| | Matrix effect ± SD | Extraction efficiency (recovery± SD) | Matrix effect ± SD | Extraction efficiency (recovery± SD) | Matrix effect ± SD | Extraction efficiency (recovery± SD) |
| **1. CBDV** | 97.96 ± 0.38 | 94.41 ± 1.36 | 96.63 ± 1.40 | 94.52 ± 0.79 | 96.78 ± 0.45 | 99.62 ± 4.23 |
| **2. CBDVA** | 101.65 ± 0.18 | 87.96 ± 5.07 | 97.63 ± 1.32 | 87.29 ± 1.09 | 97.76 ± 0.32 | 90.05 ± 3.30 |
| **3. CBG** | 86.39 ± 0.73 | 98.97 ± 1.07 | 97.98 ± 0.66 | 94.42 ± 3.89 | 94.49 ± 0.10 | 80.04 ± 2.19 |
| **4. CBD** | 96.72 ± 1.88 | 95.68 ± 1.20 | 96.11 ± 0.23 | 94.04 ± 0.50 | 96.07 ± 0.51 | 94.26 ± 5.40 |
| **5. CBDA** | 98.47 ± 0.84 | 85.05 ± 3.56 | 96.40 ± 0.82 | 87.91 ± 1.20 | 96.58 ± 0.16 | 89.70 ± 3.26 |
| **6. THCV** | 96.89 ± 0.74 | 96.55 ± 1.14 | 96.07 ± 0.63 | 94.45 ± 0.17 | 96.04 ± 0.41 | 80.73 ± 2.19 |
| **7. CBGA** | 94.14 ± 5.08 | 83.96 ± 3.02 | 96.45 ± 0.74 | 86.44 ± 1.17 | 96.30 ± 0.34 | 89.99 ± 2.99 |
| **8. CBN** | 94.82 ± 1.78 | 95.71 ± 1.19 | 93.24 ± 0.42 | 93.73 ± 0.51 | 93.90 ± 0.39 | 77.21 ± 2.18 |
| **9. $\Delta^9$-THC** | 97.11 ± 1.19 | 100.44 ± 1.13 | 95.43 ± 0.57 | 95.45 ± 0.53 | 94.60 ± 0.58 | 89.28 ± 2.29 |
| **10. $\Delta^8$-THC** | 98.13 ± 0.66 | 97.50 ± 1.00 | 95.16 ± 0.31 | 94.73 ± 0.58 | 94.97 ± 0.65 | 89.17 ± 2.61 |
| **11. THCVA** | 98.55 ± 1.22 | 82.83 ± 3.95 | 96.75 ± 1.05 | 86.49 ± 0.79 | 95.28 ± 0.57 | 89.15 ± 3.04 |
| **12. CBC** | 96.94 ± 1.54 | 98.26 ± 1.32 | 95.42 ± 0.65 | 94.46 ± 0.80 | 94.73 ± 0.19 | 79.03 ± 1.54 |
| **13.THCA** | 100.02 ± 16.37 | 98.28 ± 9.88 | 96.53 ± 2.21 | 90.27 ± 1.27 | 93.03 ± 3.22 | 88.17 ± 3.00 |
| **14. CBCA** | 99.53 ± 0.33 | 94.30 ± 0.81 | 96.63 ± 1.40 | 89.41 ± 0.72 | 93.72 ± 0.60 | 82.94 ± 1.41 |

**Supplementary Table 4. ANOVA regression statistics and LOD and LOQ for mono- and sesquiterpenoids.**

| | | Quantification Range (µg/mL) | $R^2$ | Slope ± SD | LOD (µg/mL) | LOQ (µg/mL) |
|---|---|---|---|---|---|---|
| 1. | α-Pinene | 1 - 250 | 0.9993 | 0.0043 ± 0.0001 | 0.022 | 0.065 |
| 2. | Camphene | 1 - 250 | 0.9999 | 0.0093 ± 0.0000 | 0.020 | 0.059 |
| 3. | Sabinene | 1 - 250 | 0.9996 | 0.0154 ± 0.0001 | 0.015 | 0.045 |
| 4. | β-Pinene | 1 - 250 | 0.9994 | 0.0172 ± 0.0002 | 0.018 | 0.054 |
| 5. | β-Myrcene | 1 - 250 | 0.9998 | 0.0080 ± 0.0000 | 0.014 | 0.042 |
| 6. | α-Phellandrene | 1 - 250 | 0.9998 | 0.0161 ± 0.0001 | 0.014 | 0.043 |
| 7. | $\Delta^3$-Carene | 1 - 250 | 0.9996 | 0.0038 ± 0.0000 | 0.015 | 0.044 |
| 8. | α-Terpinene | 1 - 250 | 0.9997 | 0.0094 ± 0.0001 | 0.013 | 0.038 |
| 9. | P-Cymene | 1 - 250 | 0.9997 | 0.0249 ± 0.0002 | 0.012 | 0.037 |
| 10. | Limonene | 1 - 250 | 0.9999 | 0.0069 ± 0.0000 | 0.029 | 0.088 |
| 11. | 1,8-Cineole (Eucalyptol) | 1 - 250 | 0.9991 | 0.0020 ± 0.0000 | 0.009 | 0.026 |
| 12. | Ocimene | 1 - 250 | 0.9998 | 0.0073 ± 0.0000 | 0.015 | 0.044 |
| 13. | γ-Terpinene | 1 - 250 | 1.0000 | 0.0146 ± 0.0000 | 0.012 | 0.035 |
| 14. | Sabinene Hydrate | 1 - 250 | 0.9997 | 0.0054 ± 0.0000 | 0.016 | 0.049 |
| 15. | Terpinolene | 1 - 250 | 0.9996 | 0.0081 ± 0.0001 | 0.011 | 0.033 |
| 16. | Fenchone | 1 - 250 | 0.9995 | 0.0194 ± 0.0002 | 0.012 | 0.035 |
| 17. | Linalool | 1 - 250 | 0.9996 | 0.0062 ± 0.0001 | 0.046 | 0.137 |
| 18. | Fenchol | 1 - 250 | 0.9999 | 0.0015 ± 0.0000 | 0.012 | 0.035 |
| 19. | (-)-Isopulegol | 1 - 250 | 1.0000 | 0.0023 ± 0.0000 | 0.012 | 0.037 |
| 20. | Camphor | 1 - 250 | 0.9996 | 0.0116 ± 0.0001 | 0.010 | 0.029 |
| 21. | Borneol | 1 - 250 | 0.9998 | 0.0036 ± 0.0000 | 0.026 | 0.077 |
| 22. | Terpinen-4-ol | 1 - 250 | 0.9994 | 0.0112 ± 0.0001 | 0.016 | 0.047 |
| 23. | α-Terpineol | 1 - 250 | 1.0000 | 0.0038 ± 0.0000 | 0.012 | 0.035 |
| 24. | (+)-Dihydrocarvone | 1 - 250 | 0.9999 | 0.0033 ± 0.0000 | 0.012 | 0.037 |
| 25. | Nerol | 1 - 250 | 0.9995 | 0.0111 ± 0.0001 | 0.167 | 0.500 |
| 26. | Pulegone | 1 - 250 | 0.9998 | 0.0037 ± 0.0000 | 0.014 | 0.041 |
| 27. | (+)-Carvone | 1 - 250 | 0.9999 | 0.0106 ± 0.0000 | 0.024 | 0.072 |
| 28. | Geraniol | 1 - 250 | 0.9991 | 0.0145 ± 0.0002 | 0.125 | 0.375 |
| 29. | Geranyl Acetate | 1 - 250 | 0.9996 | 0.0094 ± 0.0001 | 0.022 | 0.065 |
| 30. | (-)-β-Elemene | 1 - 250 | 0.9999 | 0.0016 ± 0.0000 | 0.017 | 0.051 |
| 31. | β-Caryophyllene | 1 - 250 | 0.9997 | 0.0021 ± 0.0000 | 0.015 | 0.045 |
| 32. | Aromadendrene | 1 - 250 | 0.9996 | 0.0020 ± 0.0000 | 0.011 | 0.033 |
| 33. | trans-β-Farnesene | 1 - 250 | 0.9992 | 0.0105 ± 0.0001 | 0.019 | 0.056 |
| 34. | α-Humulene | 1 - 250 | 0.9995 | 0.0104 ± 0.0001 | 0.013 | 0.040 |
| 35. | Valencene | 1 - 250 | 0.9996 | 0.0034 ± 0.0000 | 0.014 | 0.041 |
| 36. | Ledene | 1 - 250 | 0.9997 | 0.0014 ± 0.0000 | 0.010 | 0.029 |
| 37. | Trans-Nerolidol | 1 - 250 | 0.9995 | 0.0024 ± 0.0000 | 0.017 | 0.051 |
| 38. | Caryophyllene Oxide | 1 - 250 | 0.9997 | 0.0034± 0.0000 | 0.031 | 0.093 |
| 39. | Globulol | 1 - 250 | 0.9989 | 0.0017± 0.0000 | 0.019 | 0.057 |
| 40. | Viridiflorol | 1 - 250 | 0.9996 | 0.0020± 0.0000 | 0.020 | 0.060 |
| 41. | (-)-Guaiol | 1 - 250 | 0.9998 | 0.0043± 0.0000 | 0.018 | 0.054 |
| 42. | (+)-Cedrol | 1 - 250 | 0.9997 | 0.0029± 0.0000 | 0.017 | 0.051 |
| 43. | β-Eudesmol | 1 - 250 | 0.9997 | 0.0033± 0.0000 | 0.028 | 0.083 |
| 44. | α-Bisabolol | 1 - 250 | 0.9995 | 0.0007± 0.0000 | 0.011 | 0.033 |

**Supplementary Table 5. Trueness, precision, and accuracy for mono- and sesquiterpenoids.**

| Analyte | Spike 10 µg/mL | | | | Spike 20 µg/mL | | | | Spike 50 µg/mL | | | |
|---|---|---|---|---|---|---|---|---|---|---|---|---|
| | Repeatability – Pooled RSD% (n=3) | Intermediate precision - RSD% (n=9) | Trueness - Relative bias% (n=9) | Accuracy - Uncertainty of measurement% (n=9) | Repeatability – Pooled RSD% (n=3) | Intermediate precision - RSD% (n=9) | Trueness - Relative bias% (n=9) | Accuracy - Uncertainty of measurement% (n=9) | Repeatability – Pooled RSD% (n=3) | Intermediate precision - RSD% (n=9) | Trueness - Relative bias% (n=9) | Accuracy - Uncertainty of measurement% (n=9) |
| 1. α-Pinene | 1.5 | 4.1 | -3.9 | 5.7 | 1.6 | 2.8 | 0.9 | 2.9 | 1.8 | 4.4 | 1.3 | 4.6 |
| 2. Camphene | 1.3 | 2.0 | -0.3 | 2.0 | 1.5 | 2.4 | 3.1 | 3.9 | 1.2 | 4.0 | 2.8 | 4.9 |
| 3. Sabinene | 0.8 | 8.8 | 2.4 | 9.1 | 1.4 | 3.8 | 4.2 | 5.7 | 0.7 | 4.0 | 4.0 | 5.6 |
| 4. β-Pinene | 0.9 | 7.2 | 0.7 | 7.2 | 1.4 | 3.0 | 4.0 | 5.0 | 1.0 | 4.2 | 3.8 | 5.7 |
| 5. β-Myrcene | 0.7 | 6.5 | 4.2 | 7.7 | 1.2 | 3.3 | 6.9 | 7.6 | 0.8 | 2.8 | 5.7 | 6.3 |
| 6. α-Phellandrene | 0.7 | 5.1 | 0.5 | 5.1 | 1.4 | 2.8 | 3.4 | 4.4 | 1.1 | 3.5 | 4.0 | 5.4 |
| 7. $\Delta^3$-Carene | 0.8 | 3.4 | -0.2 | 3.4 | 1.5 | 3.2 | 4.6 | 5.6 | 1.2 | 3.6 | 5.2 | 6.3 |
| 8. α-Terpinene | 0.7 | 6.2 | 0.9 | 6.3 | 1.4 | 2.4 | 3.3 | 4.1 | 1.2 | 3.3 | 4.5 | 5.5 |
| 9. P-Cymene | 0.7 | 2.0 | -1.8 | 2.7 | 1.6 | 2.1 | 4.4 | 4.8 | 0.6 | 2.1 | 6.7 | 7.0 |
| 10. Limonene | 3.7 | 4.1 | -2.4 | 4.7 | 4.5 | 4.0 | 1.8 | 4.4 | 1.9 | 4.2 | 2.3 | 4.8 |
| 11. 1,8-Cineole(Eucalyptol) | 1.2 | 7.7 | -1.8 | 7.9 | 1.2 | 2.3 | 3.4 | 4.1 | 0.7 | 1.3 | 6.0 | 6.1 |
| 12. Ocimene | 0.5 | 2.4 | 2.0 | 3.1 | 1.3 | 1.3 | 2.8 | 3.1 | 0.5 | 2.1 | 3.0 | 3.6 |
| 13. γ-Terpinene | 0.5 | 3.4 | 1.0 | 3.6 | 1.2 | 2.3 | 2.5 | 3.4 | 0.7 | 4.2 | 2.4 | 4.9 |
| 14. Sabinene Hydrate | 1.9 | 4.4 | -0.1 | 4.4 | 3.0 | 2.7 | 6.1 | 6.7 | 4.8 | 5.9 | 3.8 | 7.0 |
| 15. Terpinolene | 0.5 | 5.0 | 0.3 | 5.1 | 1.3 | 2.0 | 3.7 | 4.2 | 0.6 | 1.3 | 5.1 | 5.2 |
| 16. Fenchone | 1.9 | 2.8 | -3.9 | 4.8 | 1.6 | 2.3 | 4.6 | 5.2 | 0.6 | 0.6 | 7.9 | 7.9 |
| 17. Linalool | 4.9 | 6.2 | 3.0 | 6.9 | 3.7 | 3.4 | 4.2 | 5.4 | 1.6 | 3.3 | 5.7 | 6.6 |
| 18. Fenchol | 1.9 | 2.8 | 4.7 | 5.4 | 1.2 | 1.1 | 7.2 | 7.3 | 0.8 | 1.0 | 8.7 | 8.8 |
| 19. (-)-Isopulegol | 3.8 | 3.7 | 1.5 | 4.0 | 1.3 | 1.8 | 6.6 | 6.8 | 1.2 | 1.2 | 7.9 | 8.0 |
| 20. Camphor | 0.8 | 4.1 | 1.7 | 4.5 | 1.2 | 2.1 | 5.7 | 6.1 | 1.3 | 3.8 | 6.2 | 7.2 |
| 21. Borneol | 1.3 | 2.7 | 1.2 | 3.0 | 1.7 | 2.3 | 2.2 | 3.2 | 1.3 | 3.4 | 4.5 | 5.6 |
| 22. Terpinen-4-ol | 3.9 | 5.8 | 2.9 | 6.5 | 1.2 | 4.1 | 5.8 | 7.2 | 1.5 | 2.9 | 4.7 | 5.5 |
| 23. α-Terpineol | 0.7 | 3.2 | 0.5 | 3.2 | 1.1 | 4.1 | 5.3 | 6.7 | 1.7 | 2.4 | 6.3 | 6.7 |
| 24. (+)-Dihydrocarvone | 1.3 | 3.8 | 0.1 | 3.8 | 1.1 | 2.7 | 3.8 | 4.7 | 1.1 | 2.2 | 6.0 | 6.4 |
| 25. Nerol | 2.6 | 3.6 | -1.5 | 3.9 | 6.4 | 6.0 | -1.3 | 6.2 | 3.6 | 3.6 | 4.5 | 5.7 |
| 26. Pulegone | 3.6 | 5.2 | 3.8 | 6.4 | 3.2 | 3.4 | 5.9 | 6.9 | 1.6 | 2.7 | 5.0 | 5.7 |
| 27. (+)-Carvone | 3.2 | 4.9 | 0.2 | 4.9 | 2.9 | 3.0 | 2.3 | 3.8 | 1.9 | 2.4 | 5.7 | 6.1 |
| 28. Geraniol | 2.0 | 5.9 | 2.3 | 6.4 | 3.5 | 5.3 | 3.1 | 6.2 | 1.2 | 2.0 | 6.2 | 6.5 |
| 29. Geranyl Acetate | 2.7 | 3.3 | 3.6 | 4.9 | 1.5 | 2.6 | 6.8 | 7.3 | 1.0 | 1.9 | 6.3 | 6.5 |
| 30. (-)-β-Elemene | 0.4 | 1.5 | -0.2 | 1.5 | 1.0 | 1.1 | 3.9 | 4.0 | 1.3 | 3.5 | 6.7 | 7.5 |
| 31. β-Caryophyllene | 0.6 | 3.1 | -4.9 | 5.8 | 0.9 | 3.4 | 3.3 | 4.7 | 0.9 | 1.1 | 6.9 | 7.0 |
| 32. Aromadendrene | 1.4 | 1.7 | -6.3 | 6.6 | 1.1 | 5.1 | 1.0 | 5.2 | 1.2 | 1.3 | 5.9 | 6.0 |
| 33. trans-β-Farnesene | 0.8 | 5.5 | 4.9 | 7.4 | 1.1 | 1.4 | 6.4 | 6.6 | 0.7 | 1.2 | 7.7 | 7.8 |
| 34. α-Humulene | 2.2 | 3.8 | -4.6 | 5.9 | 3.1 | 3.0 | 1.5 | 3.3 | 1.2 | 2.2 | 5.6 | 6.1 |
| 35. Valencene | 1.2 | 4.0 | 2.3 | 4.6 | 2.4 | 2.4 | 5.0 | 5.5 | 1.5 | 1.4 | 6.2 | 6.4 |
| 36. Ledene | 3.9 | 5.4 | -1.2 | 5.5 | 2.6 | 2.5 | 1.0 | 2.7 | 2.7 | 3.2 | 4.2 | 5.3 |
| 37. trans-Nerolidol | 2.3 | 3.9 | -1.3 | 4.1 | 3.5 | 5.5 | 1.6 | 5.7 | 2.4 | 2.4 | 5.1 | 5.7 |
| 38. Caryophyllene Oxide | 2.9 | 2.9 | 2.7 | 4.0 | 2.6 | 2.8 | 2.9 | 4.0 | 1.4 | 2.0 | 4.8 | 5.2 |
| 39. Globulol | 2.9 | 5.8 | 1.3 | 5.9 | 3.0 | 4.1 | 2.1 | 4.6 | 2.0 | 2.0 | 4.6 | 5.0 |

| | | | | | | | | | | | | | |
|---|---|---|---|---|---|---|---|---|---|---|---|---|---|
| 40. | Viridiflorol | 2.9 | 2.7 | 5.8 | 6.4 | 2.4 | 2.8 | 5.9 | 6.6 | 2.2 | 3.3 | 5.0 | 5.9 |
| 41. | (-)-Guaiol | 4.0 | 4.4 | 1.8 | 4.7 | 3.4 | 3.6 | 1.9 | 4.0 | 3.5 | 3.5 | 4.2 | 5.4 |
| 42. | (+)-Cedrol | 2.9 | 4.0 | -0.2 | 4.0 | 2.2 | 2.9 | 3.2 | 4.3 | 2.2 | 2.0 | 6.6 | 6.9 |
| 43. | β-Eudesmol | 2.5 | 3.0 | 4.4 | 5.3 | 2.6 | 4.0 | 4.1 | 5.7 | 1.7 | 1.7 | 6.5 | 6.7 |
| 44. | α-Bisabolol | 1.3 | 2.3 | 6.6 | 7.0 | 2.8 | 3.6 | 4.4 | 5.7 | 1.7 | 1.8 | 6.2 | 6.5 |

**Supplementary Table 6. ANOVA regression statistics for flavonoids.**

| | Quantification Range (µg/mL) | Correlation Coefficient $R^2$ | Slope ± SD | LOD (µg/mL) | LOQ (µg/mL) |
|---|---|---|---|---|---|
| **1. Orientin** | 1-25 | 1.0000 | 21.73 ± 0.06 | 0.04 | 0.12 |
| **2. Vitexin** | 1-25 | 1.0000 | 12.40 ± 0.03 | 0.04 | 0.11 |
| **3. Isovitexin** | 1-25 | 1.0000 | 14.99 ± 0.02 | 0.04 | 0.13 |
| **4. Quercetin** | 1-25 | 1.0000 | 14.02 ± 0.31 | 0.08 | 0.23 |
| **5. Luteolin** | 1-25 | 1.0000 | 15.76 ± 0.09 | 0.04 | 0.13 |
| **6. Kaempferol** | 1-25 | 0.9997 | 15.84 ± 0.21 | 0.07 | 0.20 |
| **7. Apigenin** | 1-25 | 1.0000 | 19.48 ± 0.07 | 0.06 | 0.17 |

## Supplementary Table 7 Accuracy (recovery) and repeatability for flavonoids in cannabis leaf over three spiked levels

| Spike level | Compound | Nominal spiked mass (µg) | Mass in sample (µg) (n=3) (mean±SD) | Measured spiked mass (µg) (n=3) (mean±SD) | Recovery% (n=3) (mean±SD) | RSD% (n=3) |
|---|---|---|---|---|---|---|
| **Level 1** | 1. Orientin | 20 | 34.27±0.04 | 17.77±0.52 | 88.8±2.6% | 2.9% |
| | 2. Vitexin | 20 | 75.24±0.08 | 21.33±0.80 | 106.6±4.0% | 3.8% |
| | 3. Isovitexin | 20 | 6.23±0.01 | 15.87±0.36 | 79.3±1.8% | 2.3% |
| | 4. Quercetin | 50 | ND | 35.73±0.67 | 71.5±1.3% | 1.9% |
| | 5. Luteolin | 50 | 58.10±0.07 | 42.03±1.74 | 84.1±3.5% | 4.1% |
| | 6. Kaempferol | 50 | ND | 36.23±0.86 | 72.5±1.7% | 2.4% |
| | 7. Apigenin | 50 | 29.50±0.03 | 40.23±0.46 | 80.5±0.9% | 1.2% |
| **Level 2** | 1. Orientin | 50 | 34.27±0.04 | 46.39±1.49 | 92.8±3.0% | 3.2% |
| | 2. Vitexin | 30 | 79.05±0.27 | 28.75±0.25 | 95.8±0.8% | 0.9% |
| | 3. Isovitexin | 50 | 6.23±0.01 | 36.25±0.76 | 72.5±1.5% | 2.1% |
| | 4. Quercetin | 100 | ND | 72.07±0.64 | 72.1±0.6% | 0.9% |
| | 5. Luteolin | 100 | 58.10±0.07 | 80.02±2.57 | 80.0±2.6% | 3.2% |
| | 6. Kaempferol | 100 | ND | 70.50±0.87 | 70.5±0.9% | 1.2% |
| | 7. Apigenin | 100 | 29.50±0.03 | 78.74±1.85 | 78.7±1.9% | 2.4% |
| **Level 3** | 1. Orientin | 80 | 34.27±0.04 | 75.78±1.34 | 94.7±1.7% | 1.8% |
| | 2. Vitexin | 80 | 75.24±0.08 | 75.60±1.64 | 94.5±2.0% | 2.2% |
| | 3. Isovitexin | 80 | 6.23±0.01 | 63.52±1.05 | 79.4±1.3% | 1.7% |
| | 4. Quercetin | 150 | ND | 115.07±3.51 | 76.7±2.3% | 3.0% |
| | 5. Luteolin | 150 | 58.10±0.07 | 121.22±2.28 | 80.8±1.5% | 1.9% |
| | 6. Kaempferol | 150 | ND | 112.67±1.10 | 75.1±0.7% | 1.0% |
| | 7. Apigenin | 150 | 29.50±0.03 | 121.69±0.85 | 81.1±0.6% | 0.7% |

## Supplementary Table 8. ANOVA regression statistics for sterols and triterpenoids.

| | Quantification Range (µg/mL) | Correlation Coefficient $R^2$ | Slope ± SD | LOD (µg/mL) | LOQ (µg/mL) |
|---|---|---|---|---|---|
| **1. Campesterol** | 1.00-100 | 0.9996 | 0.0134±0.0001 | 0.20 | 0.61 |
| **2. Stigmasterol** | 1.00-100 | 0.9999 | 0.0129±0.0001 | 0.21 | 0.63 |
| **3. β-Sitosterol** | 1.00-100 | 0.9989 | 0.0123±0.0002 | 0.26 | 0.79 |
| **4. β-Amyrin** | 1.00-100 | 0.9992 | 0.0559±0.0007 | 0.17 | 0.50 |
| **5. Epifriedelanol** | 1.00-100 | 0.9989 | 0.0280±0.0004 | 0.25 | 0.74 |
| **6. Friedelin** | 1.00-100 | 0.9998 | 0.0284±0.0002 | 0.26 | 0.78 |

## Supplementary Table 9. Trueness, precision, and accuracy for compounds for sterols and triterpenoids.

| Analyte | Spike 5 µg/mL | | | | Spike 10 µg/mL | | | | Spike 25 µg/mL | | | |
|---|---|---|---|---|---|---|---|---|---|---|---|---|
| | Repeatability - Pooled RSD% (n=3) | Intermediate precision - RSD% (n=9) | Trueness - Relative bias% (n=9) | Accuracy - Uncertainty of measurement% (n=9) | Repeatability - Pooled RSD% (n=3) | Intermediate precision - RSD% (n=9) | Trueness - Relative bias% (n=9) | Accuracy - Uncertainty of measurement% (n=9) | Repeatability - Pooled RSD% (n=3) | Intermediate precision - RSD% (n=9) | Trueness - Relative bias% (n=9) | Accuracy - Uncertainty of measurement% (n=9) |
| **1. Campesterol** | 4.9% | 4.7% | -2.6% | 5.5% | 2.3% | 2.1% | -1.1% | 2.5% | 2.3% | 2.1% | 0.5% | 2.3% |
| **2. Stigmasterol** | 4.2% | 4.7% | -4.0% | 5.8% | 4.0% | 3.9% | 0.2% | 4.0% | 1.6% | 1.4% | 0.8% | 1.8% |
| **3. β-Sitosterol** | 2.5% | 2.9% | 0.1% | 2.5% | 2.7% | 3.4% | 0.7% | 2.8% | 1.5% | 1.3% | 0.8% | 1.7% |
| **4. β-Amyrin** | 4.7% | 4.3% | -2.8% | 5.5% | 1.8% | 2.8% | -0.8% | 2.0% | 2.4% | 2.2% | 0.1% | 2.4% |
| **5. Epifriedelanol** | 3.1% | 4.5% | -2.9% | 4.3% | 1.5% | 2.2% | 0.0% | 1.5% | 1.1% | 1.1% | 0.8% | 1.4% |
| **6. Friedelin** | 3.2% | 3.0% | 0.4% | 3.3% | 2.2% | 2.9% | 1.4% | 2.7% | 3.84% | 3.6% | 0.1% | 3.8% |

## Supplementary Table 10. Cannabinoid profile in root and stem bark for three strains.

| Compound | Strain I root | Strain I stem bark | Strain II root | Strain II stem bark | Strain III root | Strain III stem bark |
|---|---|---|---|---|---|---|
| **1. CBDV** | ND | ND | ND | ND | ND | ND |
| **2. CBDVA** | ND | ND | ND | ND | ND | ND |
| **3. CBG** | 0.0007±0.00003% | 0.0002±0.00001% | ND | ND | 0.0001±0.00002% | 0.0001±0.00009% |
| **4. CBD** | ND | ND | ND | ND | ND | ND |
| **5. CBDA** | ND | ND | ND | ND | ND | ND |
| **6. THCV** | ND | ND | ND | ND | ND | ND |
| **7. CBGA** | ND | 0.0001±0.00003% | ND | 0.0001±0.00001% | 0.0002±0.00001% | 0.0001±0.00001% |
| **8. CBN** | ND | 0.0001±0.00001% | ND | ND | ND | 0.0001±0.00001% |
| **9. $\Delta^9$-THC** | 0.0001±0.00008% | 0.0006±0.00008% | ND | 0.0003±0.000001% | 0.0001±0.0001% | 0.0010±0.0002% |
| **10. $\Delta^8$-THC** | 0.0001±0.00016% | 0.0001±0.00005% | 0.0001±0.00001% | ND | ND | 0.0001±0.00004% |
| **11. THCVA** | 0.0001±0.00004% | 0.0002±0.00002% | 0.0001±0.00001% | 0.0001±0.00002% | 0.0001±0.00001% | 0.0002±0.00004% |
| **12. CBC** | ND | 0.0001±0.00001% | ND | ND | ND | 0.0001±0.000009% |
| **13.THCA** | 0.0016±0.0004% | 0.0058±0.0005% | 0.0008±0.00001% | 0.0040±0.0003% | 0.0037±0.0002% | 0.0062±0.0008% |
| **14. CBCA** | 0.0001±0.00005% | 0.0003±0.00005% | ND | 0.0003±0.000005% | 0.0001±0.00001% | 0.0004±0.00009% |
| **Total THC**** | 0.0015±0.0005% | 0.0056±0.0005% | 0.0007±0.00002% | 0.0038±0.0003% | 0.0034±0.00004% | 0.0064±0.00082% |
| **Total CBD**** | 0.0001±0.00001% | 0.0001±0.00001% | ND | ND | ND | 0.0001±0.00001% |
| **Total cannabinoids** | 0.0027±0.0006% | 0.0074±0.0008% | 0.0011±0.00005% | 0.0050±0.00007% | 0.0043±0.00007% | 0.0083±0.001% |

* Content expressed in mean ± SD% (n=3). ND=Not detected.

## Supplementary Table 11. Cannabinoid profile in leaves and inflorescences for three strains.

| Compound | Strain I leaf | Strain I inflorescence | Strain II leaf | Strain II inflorescence | Strain III leaf | Strain III inflorescence |
|---|---|---|---|---|---|---|
| **1. CBDV** | ND | ND | ND | ND | ND | ND |
| **2. CBDVA** | ND | ND | ND | ND | ND | 0.05 ± 0.003% |
| **3. CBG** | ND | 0.08 ± 0.004% | ND | 0.18 ± 0.01% | ND | 0.03 ± 0.001% |
| **4. CBD** | ND | ND | ND | ND | 0.02 ± 0.001% | 0.33 ± 0.02% |
| **5. CBDA** | ND | 0.04 ± 0.001% | ND | 0.04 ± 0.002% | 1.16 ± 0.02% | 12.06 ± 0.84% |
| **6. THCV** | ND | ND | ND | ND | ND | ND |
| **7. CBGA** | 0.02 ± 0.001%* | 0.26 ± 0.01% | 0.02 ± 0.001% | 0.37 ± 0.04% | 0.02 ± 0.001% | 0.35 ± 0.03% |
| **8. CBN** | ND | ND | ND | ND | ND | ND |
| **9. $\Delta^9$-THC** | 0.05 ± 0.002% | 0.24 ± 0.003% | 0.06 ± 0.003% | 0.30 ± 0.02% | 0.03 ± 0.001% | 0.47 ± 0.02% |
| **10. $\Delta^8$-THC** | ND | ND | ND | ND | ND | ND |
| **11. THCVA** | 0.02 ± 0.001% | 0.09 ± 0.004% | 0.01 ± 0.001% | 0.13 ± 0.007% | ND | 0.02 ± 0.001% |
| **12. CBC** | ND | ND | ND | ND | ND | 0.04 ± 0.002% |
| **13.THCA** | 1.01 ± 0.02% | 14.68 ± 0.07% | 0.68 ± 0.02% | 18.55 ± 0.70% | 0.62 ± 0.01% | 6.32 ± 0.44% |
| **14. CBCA** | 0.29 ± 0.005% | 0.39 ± 0.002% | 0.34 ± 0.01% | 0.79 ± 0.05% | 0.25 ± 0.01% | 0.24 ± 0.02% |
| **Total THC**** | 0.93 ± 0.01% | 13.11 ± 0.06% | 0.65 ± 0.02% | 16.57 ± 0.63% | 0.57 ± 0.01% | 6.02 ± 0.40% |
| **Total CBD**** | ND | 0.03 ± 0.001% | ND | 0.04 ± 0.002% | 1.04 ± 0.02% | 10.91 ± 0.75% |
| **Total cannabinoids** | 1.42 ± 0.023% | 15.77 ± 0.81% | 1.10 ± 0.04% | 20.37 ± 0.80% | 2.10 ± 0.04% | 19.93 ± 1.36% |

* Content expressed in mean (n=3) ± SD%. ND=Not detected.

** [1] Total THC = THC + 0.877× THCA, Total CBD = CBD + 0.877× CBDA

**Supplementary Table 12 Mono- and sesquiterpenoid profile in cannabis leaf and inflorescence for three cannabis strains**

| | **Compound** | **LRI Calc.** | **LRI Lit.**[a] | **Strain I Leaf** | **Strain I inflorescence** | **Strain II Leaf** | **Strain II inflorescence** | **Strain III Leaf** | **Strain III inflorescence** |
|---|---|---|---|---|---|---|---|---|---|
| 1. | α-Pinene | 934 | 932 | 0.004±0.0002%* | 0.067±0.002% | 0.002±0.0001% | 0.117±0.006% | 0.083±0.003% | 0.463±0.006% |
| 2. | Camphene | 948 | 952 | ND | 0.008±0.0001% | ND | 0.011±0.0006% | ND | ND |
| 3. | Sabinene | 974 | 976 | 0.001±0.0001% | ND | 0.001±0.0001% | ND | ND | ND |
| 4. | β-Pinene | 978 | 980 | 0.002±0.0001% | 0.047±0.001% | 0.001%±0.000% | 0.071±0.005% | 0.020±0.0007% | 0.124±0.007% |
| 5. | β-Myrcene | 992 | 992 | 0.001±0.0001% | 0.274±0.013% | ND | 0.359±0.016% | 0.024±0.0007% | 0.302±0.011% |
| 6. | α-Phellandrene | 1006 | 1006 | ND | ND | ND | ND | ND | ND |
| 7. | $\Delta^3$-Carene | 1011 | 1011 | ND | ND | ND | ND | ND | ND |
| 8. | α-Terpinene | 1017 | 1017 | ND | ND | ND | ND | ND | ND |
| 9. | P-Cymene | 1025 | 1026 | ND | ND | ND | ND | ND | ND |
| 10. | Limonene | 1029 | 1031 | ND | 0.252±0.004% | 0.004±0.0001% | 0.322±0.015% | 0.005±0.0002% | 0.049±0.002% |
| 11. | 1,8-Cineole (Eucalyptol) | 1031 | 1032 | 0.006±0.0016% | ND | 0.006±0.001% | 0.006±0.0003% | ND | ND |
| 12. | Ocimene | 1047 | 1050 | ND | 0.047±0.006% | ND | 0.045±0.003% | ND | 0.002±0.0001% |
| 13. | γ-Terpinene | 1058 | 1059 | ND | ND | ND | ND | ND | ND |
| 14. | Sabinene Hydrate | 1069 | 1068 | ND | 0.003±0.0001% | 0.001±0.0002% | 0.004±0.0005% | ND | 0.003±0.0003% |
| 15. | Terpinolene | 1089 | 1088 | ND | 0.003±0.0001% | ND | 0.003±0.0001% | ND | 0.001±0.0001% |
| 16. | Fenchone | 1088 | 1088 | ND | 0.003±0.0001% | ND | 0.003±0.0001% | ND | 0.002±0.0002% |
| 17. | Linalool | 1103 | 1100 | ND | 0.037±0.005% | 0.001±0.0001% | 0.064±0.006% | ND | 0.027±0.002% |
| 18. | Fenchol | 1117 | 1112 | ND | 0.023±0.001% | ND | 0.040±0.004% | ND | 0.009±0.0009% |
| 19. | (-)-Isopulegol | 1146 | 1146 | ND | 0.002±0.0001% | ND | 0.004±0.0005% | ND | 0.002±0.0002% |
| 20. | Camphor | 1143 | 1143 | ND | ND | ND | ND | ND | ND |
| 21. | Borneol | 1168 | 1168 | ND | 0.004±0.0002% | ND | 0.007±0.0007% | ND | 0.005±0.0004% |
| 22. | Terpinen-4-ol | 1179 | 1179 | ND | 0.001±0.0001% | ND | 0.001±0.0001% | ND | 0.001±0.0001% |
| 23. | α-Terpineol | 1194 | 1190 | ND | 0.024±0.001% | ND | 0.040±0.004% | ND | 0.013±0.0005% |
| 24. | (+)-Dihydrocarvone | 1197 | 1200 | ND | ND | ND | ND | ND | ND |
| 25. | Nerol | 1232 | 1228 | ND | ND | ND | ND | ND | ND |
| 26. | Pulegone | 1239 | 1244 | ND | ND | ND | ND | ND | ND |
| 27. | (+)-Carvone | 1245 | 1243 | ND | ND | ND | ND | ND | ND |
| 28. | Geraniol | 1258 | 1256 | ND | 0.001±0.001% | ND | 0.001±0.0002% | ND | ND |
| 29. | Geranyl Acetate | 1385 | - | ND | ND | ND | ND | ND | ND |
| | Total monoterpenoids | - | - | 0.014±0.002% | 0.796±0.008% | 0.016±0.002% | 1.097±0.045% | 0.132±0.005% | 1.004±0.043% |
| 30. | (-)-β-Elemene | 1394 | 1392 | ND | ND | ND | ND | ND | ND |
| 31. | β-Caryophyllene | 1420 | 1420 | 0.077±0.003% | 0.416±0.030% | 0.057±0.002% | 0.538±0.034% | 0.053±0.0024% | 0.086±0.002% |
| 32. | Aromadendrene | 1440 | 1440 | ND | ND | ND | ND | ND | ND |
| 33. | trans-β-Farnesene | 1459 | 1446 | 0.032±0.003% | 0.106±0.002% | 0.021±0.002% | 0.106±0.008% | 0.034±0.002% | 0.035±0.001% |
| 34. | α-Humulene | 1455 | 1455 | 0.025±0.001% | 0.123±0.006% | 0.022±0.0007% | 0.193±0.019% | 0.021±0.001% | 0.036±0.002% |
| **β - selinene** | | 1485 | 1485 | ND | 0.043±0.001% | ND | 0.046±0.001% | ND | 0.034±0.001% |
| **α - selinene** | | 1494 | 1496 | ND | 0.037±0.001% | ND | 0.036±0.001% | ND | 0.026±0.001% |
| 35. | Valencene | 1494 | 1491 | ND | ND | ND | ND | ND | 0.001±0.001% |
| 36. | Ledene | 1497 | 1493 | ND | 0.011±0.0004% | ND | 0.013±0.0009% | ND | 0.005±0.0004% |
| | **α - Farnesene** | 1508 | 1508 | ND | 0.017±0.001% | ND | 0.025±0.001% | ND | 0.024±0.001% |
| 37. | trans-Nerolidol | 1568 | 1565 | 0.012±0.003% | 0.048±0.014% | 0.009±0.0005% | 0.166±0.014% | 0.004±0.0004% | 0.009±0.001% |
| 38. | Caryophyllene Oxide | 1584 | 1583 | ND | 0.006±0.0003% | ND | 0.005±0.0008% | ND | 0.002±0.0001% |
| 39. | Globulol | 1588 | 1584 | ND | 0.002±0.0001% | ND | 0.001±0.0001% | ND | ND |
| 40. | Viridiflorol | 1595 | 1588 | ND | ND | ND | ND | ND | ND |
| 41. | (-)-Guaiol | 1602 | 1602 | ND | ND | ND | ND | 0.007±0.0003% | 0.027±0.0009% |
| 42. | (+)-Cedrol | 1605 | 1601 | ND | ND | ND | ND | ND | ND |
| 43. | β-Eudesmol | 1651 | 1650 | ND | 0.001±0.0002% | ND | 0.002±0.0001% | 0.004±0.0002% | 0.034±0.001% |
| | **α -Eudesmol** | 1653 | 1657 | ND | 0.002±0.0001% | ND | 0.002±0.0002% | 0.003±0.0002% | 0.026±0.001% |
| 44. | α-Bisabolol | 1685 | 1684 | 0.013±0.002% | 0.027±0.005% | ND | 0.002±0.0006% | 0.020±0.002% | 0.018±0.0006% |
| Total sesquiterpenoids | | - | - | 0.159±0.005% | 0.741±0.035% | 0.109±0.004% | 1.044±0.043% | 0.146±0.008% | 0.279±0.011% |
| Total mono- and sesquiterpeniods | | - | - | 0.173±0.007% | 1.537±0.043% | 0.125±0.005% | 2.141±0.102% | 0.278±0.011% | 1.283±0.018% |

* Terpenoid content expressed in mean ± SD% (n=3). Bolded terpenoid was semi-quantified by GC-FID. ND=Not detected

[a] LRI from the literature[2–4].

**Supplementary Table 13. Mono- and sesquiterpenoid ratios relative to total terpenoids in leaves and inflorescences**

| Compound | | LRI Calc. | LRI Lit.[a] | Strain I Leaf | Strain I inflorescence | Strain II Leaf | Strain II inflorescence | Strain III Leaf | Strain III inflorescence |
|---|---|---|---|---|---|---|---|---|---|
| 1. | α-Pinene | 934 | 932 | 2.37±0.22%* | 4.36±0.04% | 1.82±0.07% | 5.46±0.17% | 29.81±0.75% | 36.07±0.84% |
| 2. | Camphene | 948 | 952 | 0.18±0.015% | 0.51±0.01% | 0.17±0.01% | 0.51±0.02% | ND | ND |
| 3. | Sabinene | 974 | 976 | 0.30±0.05% | ND | 0.56±0.05% | ND | ND | ND |
| 4. | β-Pinene | 978 | 980 | 1.08±0.06% | 3.04±0.02% | 0.89±0.02% | 3.31±0.13% | 7.12±0.16% | 9.67±0.16% |
| 5. | β-Myrcene | 992 | 992 | 0.52±0.07% | 17.82±0.65% | ND | 16.78±0.99% | 8.74±0.16% | 23.57±0.08% |
| 6. | α-Phellandrene | 1006 | 1006 | ND | ND | ND | ND | ND | ND |
| 7. | $\Delta^3$-Carene | 1011 | 1011 | ND | ND | 0.21±0.05% | ND | ND | ND |
| 8. | α-Terpinene | 1017 | 1017 | 0.11±0.02% | ND | ND | ND | ND | ND |
| 9. | P-Cymene | 1025 | 1026 | ND | ND | 0.23±0.03% | ND | ND | ND |
| 10. | Limonene | 1029 | 1031 | ND | 16.42±0.59% | 0.74±0.14% | 15.03±0.62% | 1.77±0.06% | 3.79±0.04% |
| 11. | 1,8-Cineole (Eucalyptol) | 1031 | 1032 | 3.19±0.80% | ND | 4.56±0.73% | 0.28±0.04% | ND | 0.03±0.01% |
| 12. | Ocimene | 1047 | 1050 | ND | 3.07±0.32% | ND | 2.08±0.04% | ND | 0.19±0.01% |
| 13. | γ-Terpinene | 1058 | 1059 | 0.14±0.01% | ND | 0.23±0.03% | ND | ND | ND |
| 14. | Sabinene Hydrate | 1069 | 1068 | ND | 0.22±0.01% | 0.74±0.14% | 0.02±10.57% | ND | 0.27±0.02% |
| 15. | Terpinolene | 1089 | 1088 | ND | 0.16±0.01% | ND | 0.15±0.01% | ND | 0.07±0.001% |
| 16. | Fenchone | 1088 | 1088 | ND | 0.22±0.001% | ND | 0.16±0.001% | ND | 0.13±0.01% |
| 17. | Linalool | 1103 | 1100 | ND | 2.41±0.07% | ND | 2.99±0.19% | ND | 2.10±0.21% |
| 18. | Fenchol | 1117 | 1112 | ND | 1.46±0.01% | ND | 1.85±0.11% | ND | 0.72±0.06% |
| 19. | (-)-Isopulegol | 1146 | 1146 | ND | 0.15±0.001% | ND | 0.17±0.01% | ND | 0.12±0.01% |
| 20. | Camphor | 1143 | 1143 | ND | ND | ND | ND | ND | ND |
| 21. | Borneol | 1168 | 1168 | ND | 0.29±0.01% | ND | 0.32±0.02% | ND | 0.40±0.02% |
| 22. | Terpinen-4-ol | 1179 | 1179 | ND | 0.06±0.001% | ND | 0.05±0.001% | ND | 0.06±0.01% |
| 23. | α-Terpineol | 1194 | 1190 | ND | 1.56±0.05% | ND | 1.86±0.15% | ND | 1.04±0.03% |
| 24. | (+)-Dihydrocarvone | 1197 | 1200 | ND | ND | ND | ND | ND | ND |
| 25. | Nerol | 1232 | 1228 | ND | ND | ND | ND | ND | ND |
| 26. | Pulegone | 1239 | 1244 | ND | ND | ND | ND | ND | ND |
| 27. | (+)-Carvone | 1245 | 1243 | ND | ND | ND | ND | ND | 0.02±0.001% |
| 28. | Geraniol | 1258 | 1256 | ND | 0.04±0.001% | ND | 0.05±0.001% | ND | ND |
| 29. | Geranyl Acetate | 1385 | - | ND | ND | ND | ND | ND | ND |
| Total monoterpenoids | | - | - | 7.91±0.72% | 51.8±0.94% | 12.93±0.82% | 51.25±1.55% | 47.45±1.12% | 78.25±0.65% |
| 30. | (-)-β-Elemene | 1394 | 1392 | ND | ND | ND | ND | ND | ND |
| 31. | β-Caryophyllene | 1420 | 1420 | 44.61±0.74% | 27.09±0.35% | 45.25±0.78% | 25.14±0.70% | 19.25±0.34% | 6.71±0.19% |
| 32. | Aromadendrene | 1440 | 1440 | ND | ND | ND | ND | ND | ND |
| 33. | trans-β-Farnesene | 1459 | 1446 | 18.75±2.23% | 6.87±0.09% | 17.12±1.3% | 4.96±0.31% | 12.17±0.06% | 2.73±0.05% |
| 34. | α-Humulene | 1455 | 1455 | 14.22±0.19% | 7.97±0.18% | 17.34±0.85% | 9.01±0.52% | 7.68±0.22% | 2.82±0.09% |
| | **β - selinene** | 1485 | 1485 | ND | 0.04±0.001% | ND | 0.05±0.001% | ND | 0.03±0.001% |
| | **α - selinene** | 1494 | 1496 | ND | 0.04±0.001% | ND | 0.04±0.001% | ND | 0.03±0.001% |
| 35. | Valencene | 1494 | 1491 | ND | ND | ND | ND | ND | 0.06±0.001% |
| 36. | Ledene | 1497 | 1493 | ND | 0.74±0.01% | ND | 0.63±0.03% | ND | 0.39±0.03% |
| | **α - Farnesene** | 1508 | 1508 | ND | 0.02±0.01% | ND | 0.03±0.001% | ND | 0.02±0.001% |
| 37. | trans-Nerolidol | 1568 | 1565 | 7.07±1.51% | 3.11±0.46% | 7.35±0.38% | 7.73±0.64% | 1.49±0.14% | 0.67±0.09% |
| 38. | Caryophyllene Oxide | 1584 | 1583 | ND | 0.38±0.01% | ND | 0.25±0.02% | ND | 0.16±0.01% |
| 39. | Globulol | 1588 | 1584 | ND | 0.10±0.001% | ND | 0.06±0.001% | ND | ND |
| 40. | Viridiflorol | 1595 | 1588 | ND | ND | ND | ND | ND | ND |
| 41. | (-)-Guaiol | 1602 | 1602 | ND | ND | ND | ND | 2.36±0.05% | 2.12±0.05% |
| 42. | (+)-Cedrol | 1605 | 1601 | ND | ND | ND | ND | ND | 0.02±0.001% |
| 43. | β-Eudesmol | 1651 | 1650 | ND | 0.07±0.01% | ND | 0.07±0.02% | 1.50±0.04% | 2.64±0.11% |
| | **α -Eudesmol** | 1653 | 1657 | ND | 0.10±0.02% | ND | 0.08±0.01% | 0.90±0.09% | 2.01±0.10% |
| 44. | α-Bisabolol | 1685 | 1684 | 7.42±0.65% | 1.76±0.01% | ND | 0.82±0.11% | 7.20±0.63% | 1.42±0.09% |
| Total sesquiterpenoids | | - | - | 92.08±0.72% | 48.20±0.94% | 87.07±0.82% | 48.75±1.55% | 52.55±1.12% | 21.75±0.65% |
| Total mono- and sesquiterpenoids | | - | - | 100% | 100% | 100% | 100% | 100% | 100% |

* Terpenoid ratio (relative to total terpenoid) is expressed as mean value ± SD% (n=3). ND= Not detected. Bolded entries were semi-quantified by relative area by GC-FID.

[a] LRI from the literature[2–4].

**Supplementary Table 14** Flavonoid profile in cannabis

| Compound | Orientin | Vitexin | Isovitexin | Quercetin | Luteolin | Kaempferol | Apigenin | Total flavonoids |
|---|---|---|---|---|---|---|---|---|
| Strain I leaf | 0.08± 0.003% | 0.12± 0.008% | 0.01± 0.004% | ND | 0.09± 0.008% | ND | 0.05± 0.004% | 0.34± 0.02% |
| Strain II leaf | 0.07± 0.002% | 0.17± 0.005% | 0.02± 0.002% | ND | 0.10± 0.01% | ND | 0.07± 0.006% | 0.44± 0.02% |
| Strain III leaf | 0.07± 0.002% | 0.13± 0.004% | 0.05± 0.001% | ND | 0.05± 0.002% | ND | 0.03± 0.001% | 0.40± 0.009% |
| Strain I inflorescence | 0.01± 0.0003% | 0.02± 0.0006% | 0.002± 0.00005% | 0.01± 0.0003% | 0.01± 0.0004% | 0.01± 0.0002% | 0.004± 0.0002% | 0.07± 0.001% |
| Strain II inflorescence | 0.02± 0.001% | 0.03± 0.002% | 0.002± 0.0001% | 0.01± 0.0004% | 0.02± 0.001% | 0.01± 0.0002% | 0.01± 0.0004% | 0.10± 0.005% |
| Strain III inflorescence | 0.03± 0.0001% | 0.06± 0.0008% | 0.01± 0.0001% | 0.01± 0.0008% | 0.02± 0.0001% | 0.005± 0.0003% | 0.01± 0.0003% | 0.14± 0.002% |

* Content expressed in mean ± SD% (n=3). ND = Not detected.

**Supplementary Table 15** Sterol profile in cannabis

| Compound | Campesterol | Stigmasterol | β-sitosterol | Total sterols |
|---|---|---|---|---|
| Strain I root | 0.016 ± 0.001% | 0.010 ± 0.001% | 0.051 ± 0.004% | 0.077 ± 0.005% |
| Strain II root | 0.020 ± 0.001% | 0.009 ± 0.0005% | 0.058 ± 0.003% | 0.088 ± 0.004% |
| Strain III root | 0.010 ± 0.0004% | 0.013 ± 0.001% | 0.040 ± 0.001% | 0.063 ± 0.002% |
| Strain I stem bark | 0.016 ± 0.001% | 0.011 ± 0.0003% | 0.044 ± 0.002% | 0.071 ± 0.003% |
| Strain II stem bark | 0.016 ± 0.001% | 0.008 ± 0.0004% | 0.044 ± 0.003% | 0.069 ± 0.004% |
| Strain III stem bark | 0.017 ± 0.0005% | 0.010 ± 0.001% | 0.050 ± 0.007% | 0.076 ± 0.006% |
| Strain I leaf | ND | 0.026 ± 0.003% | 0.027 ± 0.001% | 0.053 ± 0.003% |
| Strain II leaf | ND | 0.030 ± 0.002% | 0.022 ± 0.0004% | 0.052 ± 0.002% |
| Strain III leaf | ND | 0.030 ± 0.001% | 0.024 ± 0.0002% | 0.053 ± 0.001% |

* Content expressed in mean ± SD% (n=3). ND = Not detected.

**Supplementary Table 16** Triterpenoid profile in cannabis

| Compound | β-Amyrin | Epifriedelanol | Friedelin | Total triterpenoids |
|---|---|---|---|---|
| Strain I root | 0.006 ± 0.0003% | 0.043 ± 0.004% | 0.083 ± 0.007% | 0.132 ± 0.011% |
| Strain II root | 0.005 ± 0.0001% | 0.033 ± 0.001% | 0.091 ± 0.004% | 0.128 ± 0.005% |
| Strain III root | 0.013 ± 0.001% | 0.092 ± 0.003% | 0.135 ± 0.003% | 0.239 ± 0.006% |
| Strain I stem bark | 0.006 ± 0.001% | 0.013 ± 0.001% | 0.033 ± 0.004% | 0.052 ± 0.006% |
| Strain II stem bark | 0.006 ± 0.001% | 0.021 ± 0.002% | 0.065 ± 0.007% | 0.092 ± 0.010% |
| Strain III stem bark | 0.007 ± 0.0004% | 0.041 ± 0.002% | 0.100 ± 0.004% | 0.149 ± 0.007% |
| Strain I leaf | 0.026 ± 0.002% | ND | ND | 0.026 ± 0.002% |
| Strain II leaf | 0.024 ± 0.001% | ND | ND | 0.024 ± 0.001% |
| Strain III leaf | 0.012 ± 0.001% | ND | ND | 0.012 ± 0.001% |

* Content expressed in mean ± SD% (n=3). ND = Not detected.

**Supplementary Table 17. Chemical profile of cannabis plant parts**

| | Root | Stem bark | Leaf | Inflorescence |
|---|---|---|---|---|
| **Total cannabinoids** | -* | - | 1.10% - 2.10% | 15.77% - 20.37% |
| **Total mono- and sesquiterpenoids** | - | - | 0.13% - 0.28% | 1.28% - 2.14% |
| **Total triterpenoids** | 0.13% - 0.24% | 0.05% - 0.15% | - | - |
| **Total sterols** | 0.06% - 0.09% | 0.07 - 0.08% | 0.05% - 0.05% | - |
| **Total flavonoids** | - | - | 0.34% - 0.44% | 0.07% - 0.14% |

* Less than 0.05%.

**Supplementary Table 18.** SIM method parameters for cannabinoids

| Name | Retention Time (min) | POS/NEG | Quantifier |
|---|---|---|---|
| **IS: Δ$^{9}$-THC-*d*$_{3}$** | 16.059 | POS | 318.3 |
| **1. CBDV** | 8.102 | POS | 287.2 |
| **2. CBDVA** | 9.256 | POS | 331.2 |
| **3. CBG** | 11.263 | POS | 317.3 |
| **4. CBD** | 11.410 | POS | 315.3 |
| **5. CBDA** | 12.181 | POS | 359.2 |
| **6. THCV** | 12.393 | POS | 287.2 |
| **7. CBGA** | 13.539 | POS | 343.3 |
| **8. CBN** | 15.048 | POS | 311.2 |
| **9. Δ$^{9}$-THC** | 16.471 | POS | 315.3 |
| **10. Δ$^{8}$-THC** | 17.156 | POS | 315.3 |
| **11. THCVA** | 17.282 | POS | 331.2 |
| **12. CBC** | 18.398 | POS | 315.2 |
| **13.THCA** | 22.350 | POS | 359.3 |
| **14. CBCA** | 23.722 | POS | 359.3 |

**Supplementary Table 19.** SIM method parameters for flavonoids

| Name | Retention Time (min) | POS/NEG | Quantifier |
|---|---|---|---|
| 1. Orientin | 15.393 | NEG | 447 |
| 2. Vitexin | 19.444 | NEG | 431 |
| 3. Isovitexin | 21.643 | NEG | 431 |
| 4. Quercetin | 36.288 | NEG | 301 |
| 5. Luteolin | 40.932 | NEG | 285 |
| 6. Kaempferol | 44.875 | NEG | 285 |
| 7. Apigenin | 48.708 | NEG | 269 |

**Supplementary Table 20.** SIM method parameters for monoterpenoids and sesquiterpenoids

| | Retention Time (min) | Quantifier | Qualifier 1 | Qualifier 2 | Qualifier 3 |
|---|---|---|---|---|---|
| IS: tridecane | 11.497 | 71 | 85 | 57 | |
| 1. α-Pinene | 5.597 | 77 | 91 | 121 | |
| 2. Camphene | 5.787 | 93 | 121 | 136 | |
| 3. Sabinene | 6.073 | 93 | 77 | 79 | 136 |
| 4. β-Pinene | 6.132 | 93 | 69 | 77 | 136 |
| 5. β-Myrcene | 6.242 | 69 | 79 | 136 | |
| 6. α-Phellandrene | 6.462 | 93 | 77 | 91 | 136 |
| 7. $\Delta^3$-Carene | 6.542 | 77 | 91 | 121 | |
| 8. α-Terpinene | 6.621 | 121 | 91 | 93 | 136 |
| 9. *p*-Cymene | 6.732 | 119 | 91 | 134 | |
| 10. Limonene | 6.790 | 93 | 121 | 136 | |
| 11. 1,8-Cineole (Eucalyptol) | 6.835 | 154 | 93 | 139 | |
| 12. Ocimene | 7.004 | 93 | 79 | 121 | |
| 13. γ-Terpinene | 7.207 | 93 | 77 | 121 | 136 |
| 14. Sabinene Hydrate | 7.373 | 71 | 93 | 121 | 154 |
| 15. Terpinolene | 7.662 | 93 | 121 | 136 | |
| 16. Fenchone | 7.685 | 81 | 69 | 152 | |
| 17. Linalool | 7.816 | 93 | 69 | 121 | |
| 18. Fenchol | 8.145 | 93 | 111 | 121 | |
| 19. (-)-Isopulegol | 8.656 | 121 | 136 | 154 | |
| 20. Camphor | 8.646 | 95 | 108 | 152 | |
| 21. Borneol | 9.064 | 110 | 67 | 139 | |
| 22. Terpinen-4-ol | 9.238 | 71 | 93 | 111 | 154 |
| 23. α-Terpineol | 9.492 | 121 | 59 | 136 | |
| 24. (+)-Dihydrocarvone | 9.569 | 95 | 109 | 137 | 152 |
| 25. Nerol | 10.120 | 69 | 93 | 121 | |
| 26. Pulegone | 10.398 | 109 | 67 | 152 | |
| 27. (+)-Carvone | 10.485 | 82 | 93 | 108 | |
| 28. Geraniol | 10.613 | 69 | 93 | 123 | |
| 29. Geranyl Acetate | 13.309 | 69 | 93 | 121 | |
| 30. (-)-β-Elemene | 13.665 | 147 | 161 | 189 | |
| 31. β-Caryophyllene | 14.339 | 105 | 91 | 79 | |
| 32. Aromadendrene | 14.775 | 161 | 189 | 204 | |
| 33. trans-β-Farnesene | 14.988 | 69 | 93 | 133 | |
| 34. α-Humulene | 15.100 | 93 | 79 | 91 | |
| 35. Valencene | 15.974 | 161 | 189 | 204 | |
| 36. Ledene | 16.000 | 161 | 189 | 204 | |
| 37. trans-Nerolidol | 17.406 | 93 | 107 | 136 | |
| 38. Caryophyllene Oxide | 17.851 | 95 | 105 | 107 | |
| 39. Globulol | 17.889 | 161 | 189 | 204 | |
| 40. Viridiflorol | 18.021 | 161 | 189 | 204 | |
| 41. (-)-Guaiol | 18.067 | 161 | 105 | 107 | |
| 42. (+)-Cedrol | 18.192 | 150 | 95 | 151 | |
| 43. β-Eudesmol | 18.810 | 59 | 149 | 164 | |
| 44. α-Bisabolol | 19.135 | 204 | 189 | 161 | |

**Supplementary Table 21.** SIM method parameters for triterpenoids and sterols

| Name | Retention Time (min) | Quantifier | Qualifier 1 | Qualifier 2 |
|---|---|---|---|---|
| IS: Cholesterol | 16.093 | 105 | 133 | 386 |
| 1. Campesterol | 17.044 | 105 | 133 | 400 |
| 2. Stigmasterol | 17.350 | 105 | 133 | 412 |
| 3. β-Sitosterol | 17.955 | 105 | 133 | 414 |
| 4. β-Amyrin | 18.466 | 218 | 189 | 203 |
| 5. Epifriedelanol | 20.519 | 109 | 123 | 413 |
| 6. Friedelin | 20.900 | 109 | 123 | 426 |

**References**


1. ElSohly, M. A. & Gul, W. Constituents of Cannabis Sativa. in *Handbook of Cannabis* 3–22 (Oxford University Press, 2014). doi:10.1093/acprof:oso/9780199662685.003.0001.
2. Adams, R. P. The serrate leaf margined Juniperus (Section Sabina) of the western hemisphere: systematics and evolution based on leaf essential oils and Random Amplified Polymorphic DNAs (RAPDs). *Biochemical systematics and ecology* **28**, 975–989 (2000).
3. Lucero, M. E., Estell, R. E. & Fredrickson, E. L. The Essential Oil Composition of Psorothamnus scoparius (A. Gray) Rydb. *Journal of Essential Oil Research* **15**, 108–111 (2003).
4. Linstrom, P. F. & Mallard, W. *NIST Chemistry WebBook-SRD 69*. (2001).
